# Post-Kohn–Sham Random-Phase Approximation and Correction Terms in the Expectation-Value Coupled-Cluster Formulation

**DOI:** 10.1021/acs.jctc.3c00496

**Published:** 2023-09-29

**Authors:** Dominik Cieśliński, Aleksandra M. Tucholska, Marcin Modrzejewski

**Affiliations:** † Faculty of Chemistry, University of Warsaw, Pasteura 1, Warsaw 02-093, Poland; ‡ Institute of Physics, Łódź University of Technology, Wólczańska 219, 90-924 Łódź, Poland

## Abstract

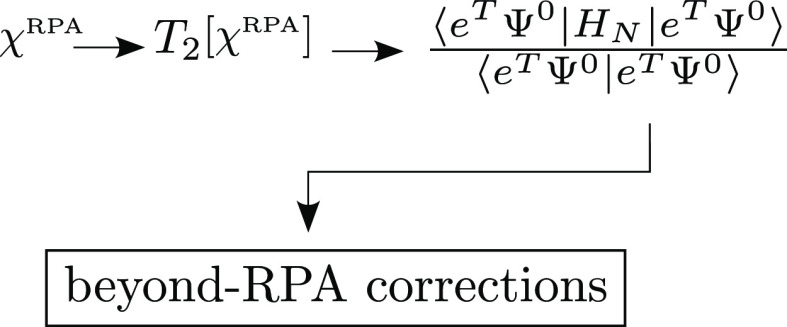

Using expectation-value coupled-cluster theory and many-body perturbation theory (MBPT), we formulate a series of corrections to the post-Kohn–Sham (post-KS) random-phase approximation (RPA) energy. The beyond-RPA terms are of two types: those accounting for the non-Hartree–Fock reference and those introducing the coupled-cluster doubles non-ring contractions. The contributions of the former type, introduced via the semicanonical orbital basis, drastically reduce the binding strength in noncovalent systems. The good accuracy is recovered by the attractive third-order doubles correction referred to as *E*_c_^2*g*^. The existing RPA approaches based on KS orbitals neglect most of the proposed corrections but can perform well thanks to error cancellation. The proposed method accounts for every contribution in the state-of-the-art renormalized second-order perturbation theory (rPT2) approach but adds additional terms which initially contribute in the third order of MBPT. The cost of energy evaluation scales as noniterative  in the implementation with low-rank tensor decomposition. The numerical tests of the proposed approach demonstrate accurate results for noncovalent dimers of polar molecules and for the challenging many-body noncovalent cluster of CH_4_···(H_2_O)_20_.

## 1 Introduction

The random-phase approximation (RPA), or the ring-diagram approximation, originated in electron gas physics as a method developed by, among others, Gell-Mann and Brueckner to sum up the most divergent correlation energy terms in the high-density regime.^1,2^ In the single-reference quantum chemical methodology, RPA is usually understood as a post-Kohn–Sham (post-KS) method where the KS orbitals from some density functional theory (DFT) approximation are used to build the RPA interacting density-response function χ^RPA^.^3–6^

The RPA response is the central quantity used to formulate the correlation energy approximation via the adiabatic-connection fluctuation–dissipation formula
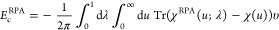
1where the integration is over the coupling constant λ and frequency *u*. It turns out that RPA in eq 1 is enough to capture the essential dispersion forces in many-body noncovalent systems.^5,7–9^ At long intermolecular distances, the RPA correlation energy correctly reduces to the Casimir–Polder dispersion energy with correlated response functions of the monomers.^5,10^ χ^RPA^ accounts for the electrodynamic screening in large polarizable noncovalent complexes, which is a neglected effect in, e.g., second-order Møller–Plesset theory.^11,12^ In molecular crystals and finite clusters, post-KS RPA reproduces the nonadditive many-body effects at a qualitative level, which is beyond the reach of local DFT approximations.^13–17^

Still, there are some obvious limitations of RPA, e.g., the lack of single excitations and the presence of exclusion-principle violating terms. Within DFT, a way to improve RPA is to use an ab initio Kohn–Sham Hamiltonian^18–20^ and to combine it with a more sophisticated model of the exchange–correlation kernel in the screening equation.^21,22^ An alternative, which we consider in this work, is to apply many-body perturbation theory (MBPT) and coupled-cluster techniques to account for the beyond-RPA contributions. The state-of-the-art approach of this type is the renormalized second-order perturbation theory (rPT2) method of Ren et al.,^23^ where *E*_c_^RPA^ is supplemented with the renormalized singles energy, *E*_c_^rSE^, and the second-order screened exchange energy,^24^*E*_c_^SOSEX^.

Both *E*_c_^rSE^ and *E*_c_^SOSEX^ are well-justified in MBPT. The singles energy accounts for a subset of terms that arise for the non-Hartree–Fock reference. The second-order screened exchange (SOSEX) energy removes the second-order Pauli-exclusion violating contributions in *E*_c_^RPA^.^25^ However, using both of those corrections at the same time leads to a systematic overbinding in hydrogen-bonded systems.^23^ For this reason, the simple RPA plus singles approach remains the most popular RPA method for noncovalent systems.^23,26–28^

The goal of our work is to develop a systematic approach where a number of third- and higher-order terms beyond the rPT2 energy are included. The work is organized as follows. We start from the correlation energy expressed as the expectation value of the normal-ordered Hamiltonian, *H*_N_, with contributions evaluated directly via the one-electron reduced density matrix (1-RDM) and the two-electron reduced density matrix (2-RDM) cumulant. The density matrices are then parametrized using the RPA doubles amplitudes, *T*_2_, and the mean-field approximation of 1-RDM. This leads to an expansion where the leading term is the conventional RPA correlation energy, followed by the singles and non-ring doubles corrections. Among the derived terms, we select a subset of contributions with the  scaling. In the numerical section, we test the proposed approach for noncovalent systems: the dispersion-dominated and hydrogen-bonded dimers of the S66 × 8 data set^29^ and the many-body cluster of the methane molecule in a dodecahedral water cage.^13^

## 2 Theory

### 2.1 Definitions

We assume a closed-shell system with real orbitals, but an open-shell generalization of the presented equations is possible. The naming of the indices is as follows.

- Occupied (spin)orbitals: *i*, *j*, *k*, *l*
- Virtual (spin)orbitals: *a*, *b*, *c*, *d*
- General (spin)orbitals: *p*, *q*, *r*, *s*
- Eigenvectors of the RPA doubles amplitudes: κ, μ, ν
- Tensor hypercontraction vectors^30^ (grid points): γ, δ

We distinguish between spin orbital and orbital indices by the label above the summation symbol, *∑*^spin-orb^ and *∑*^orb^. The two electron integrals (*pq*|*rs*) are defined as
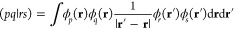
2Where applicable, the RPA amplitudes, *T*_*ij*_^*ab*^, are treated as matrices with compound indices *ai* and *bj*. The κth eigenvector of this matrix is denoted as *U*_*ai*,κ_. The matrix product involving *T*_2_ and, for example, the Coulomb integral matrix *V*, is defined as
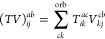
3

The direct-ring approximation of an algebraic formula  is understood as the explicit part of  which can be expressed with the matrix–matrix multiplication defined in eq 3 or, equivalently, can be graphically represented by ring diagrams with no exchange interactions. The MBPT and coupled-cluster diagrams follow the left–right convention described in ref (31).

The total electronic Hamiltonian, *H*, is used in the normal-ordered form. Thus, *H* is the sum
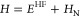
4where *E*^HF^ is the reference mean-field energy
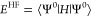
5and *H*_N_ is the normal-ordered part of *H*
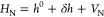
6

The above decomposition of *H*_N_, with the zeroth-order part *h*^0^ and perturbation δ*h* + *V*_N_, defines the MBPT contributions. The zeroth-order mean-field Hamiltonian
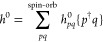
7has the single-determinant ground state |Ψ^0^⟩, which defines the split into the occupied and virtual orbital subspaces. The normal ordering of operators with respect to |Ψ^0^⟩ is indicated by curly braces. *h*^0^ can be any mean-field model. Here, we will consider the Kohn–Sham Hamiltonian in the PBE exchange–correlation approximation^32^ and the generalized many-body theory (GMBPT) Hamiltonian^33^ based on PBE. The perturbation in eq 6 is composed of the one-electron part
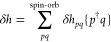
8and the two-electron part in the form of the normal-ordered Coulomb interaction operator
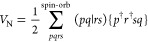
9

The one-electron perturbation originates from the difference
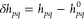
10between the matrix elements of the Fock matrix
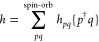
11i.e., the one-electron part of *H*_N_, and the elements of the reference mean-field Hamiltonian, *h*^0^. The Fock matrix *h* is computed using the 1-RDM, ρ^0^, corresponding to |Ψ^0^⟩.

### 2.2 Decomposition of the Correlation Energy

Let us consider the correlation energy relative to the reference determinant |Ψ^0^⟩, defined as the expectation value of the normal-ordered Hamiltonian with the interacting wave function |Ψ⟩
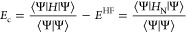
12

A direct evaluation of eq 12 requires a model of 1-RDM
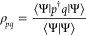
13and 2-RDM
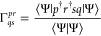
14

To arrive at a useful truncation scheme, we split Γ into the antisymmetrized product of 1-RDMs and the size-extensive remainder, Λ, referred to as the 2-RDM cumulant^34–36^
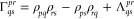
15

Using the above definitions and Wick’s theorem, the complete matrix element of *H*_*N*_ on the rhs of eq 12 can be built from the one-particle contributions
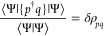
16and the two-particle contributions
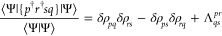
17where δρ is the difference between the fully interacting 1-RDM and the diagonal, noninteracting density matrix, ρ^0^, corresponding to the single determinant |Ψ^0^⟩
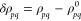
18

Combining eqs 16, 17, and 12 yields the decomposition of the total correlation energy into
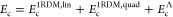
19

The total 1-RDM contribution, *E*_c_^1RDM^ = *E*_c_^1RDM,lin^ + *E*_c_^1RDM,quad^, is composed of the linear component, which originates from the single-particle part of *H*_N_
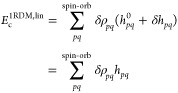
20and the quadratic part, which originates from the trace of *V* with the antisymmetrized product of 1-RDMs
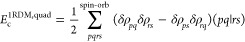
21

The cumulant part of the correlation energy, *E*_c_^Λ^, is the trace of *V* with the cumulant matrix
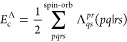
22

The working equations for *E*_c_^1RDM,lin^, *E*_c_^1RDM,quad^, and *E*_c_^Λ^ require parametrization of the wave function. In what follows, we will assume that the reduced density matrices originate from the coupled-cluster doubles (CCD) wave function
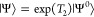
23in which the cluster operator, *T*_2_, accounts only for the ring (RPA) terms.^37^ Infinite-order ring formulas for ρ and Λ follow from the expectation-value coupled-cluster theory.^38^ In addition to the CCD contribution, the effect of single excitations, which is the leading-order contribution to *E*_c_^1RDM,lin^ and *E*_c_^1RDM,quad^, will be accounted for by using mean-field 1-RDMs built from the eigenvectors of the Fock matrix. We will use the terms “singles correction”, “mean-field change energy”, and “1-RDM energy” interchangeably for *E*_c_^1RDM^.

### 2.3 Choice of *h*^0^

In traditional post-KS RPA, the noninteracting system is described by the KS Hamiltonian *h*^KS^; the one-particle perturbation is then
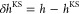
24where *h* is the Fock matrix, as in the Hartree–Fock theory, but computed with KS orbitals. However, as noted by Bartlett et al.,^33,39,40^ δ*h*^KS^ includes large diagonal matrix elements which make finite-order perturbation theory poorly behaved. GMBPT, employed throughout this work, addresses this issue.^39^

In GMBPT,^39^ the occupied–occupied (oo) and virtual–virtual (vv) blocks of the one-particle perturbation are combined into the zeroth-order Hamiltonian defined as^18,33^
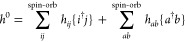
25

The GMBPT perturbation, δ*h* = *h* – *h*^0^, is limited to the remaining ov and vo blocks of the Fock matrix.

The occupied and virtual orbital subspaces are still defined by the underlying KS Hamiltonian, but the KS orbitals and orbital energies in all MBPT formulas are replaced by their semicanonical counterparts. The semicanonical orbitals, *C*, and orbital energies, ε, are the eigenvectors and eigenvalues of the oo and vv blocks, respectively, of the Fock matrix
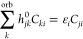
26
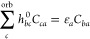
27

In the semicanonical basis, *h*^0^ is diagonal
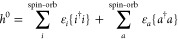
28and the one-electron perturbation δ*h* has the form
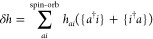
29

The semicanonical basis has been originally applied by Verma and Bartlett in their implementation of self-consistent RPA.^18^

From the MBPT perspective, the justification of using the semicanonical basis for the non-Hartree–Fock mean-field reference is clear. Take for instance the second-order RPA correlation term
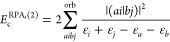
30

The semicanonical-orbital *E*_c_^RPA,(2)^ is equivalent to the infinite sum of the KS-orbital terms with perturbation δ*h*^KS^, analogous to diagram V in Table 1 (see also Section 4 of the Supporting Information). Only the first term in the series is included in the traditional RPA approach based on the KS orbitals. From now on, we assume that all of the orbitals and orbital energies are semicanonical. The KS orbitals will be used only in numerical comparisons to obtain the results of the existing RPA approaches.

**Table 1:**
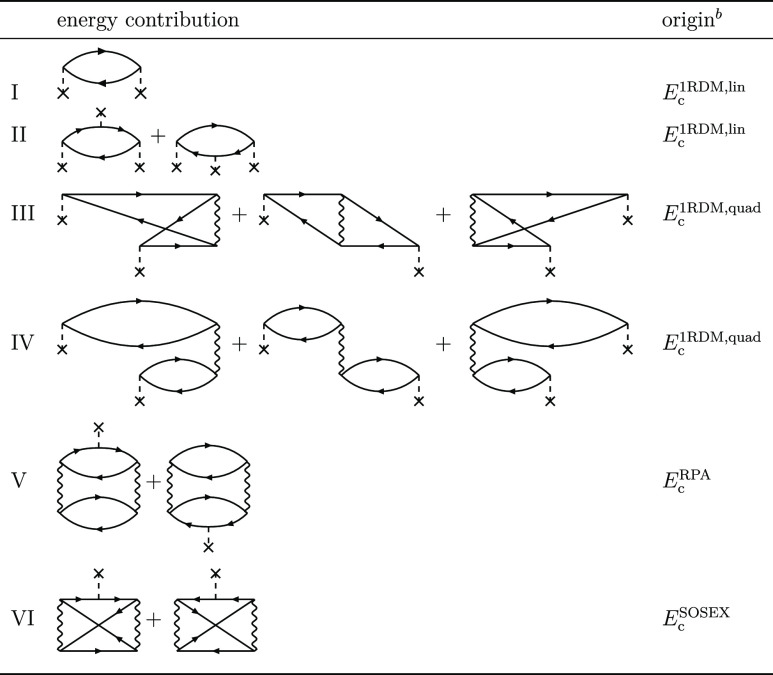
Subset of Second- and Third-Order Correlation Energy Diagrams Which Include the One-Particle Perturbation^a^^c^

a The rightmost column indicates the contributing term in the implemented variant of the correlation energy.

b Energy components indicated as the origin contribute terms I–VI with the exact prefactors as implied by the diagram translation rules.

c Operator depicted as “X” is the one-particle perturbation δ*h*^KS^ = *h* – *h*^KS^. Note that in the semicanonical basis, the diagonal blocks of the one-particle perturbation are zero. See Figures 14a–d of ref (31) for the complete set of third-order diagrams.

### 2.4 RPA Doubles Amplitudes

The RPA double-excitation amplitude matrix, *T*_2_, is a key intermediate replacing the infinite sums of ring contributions in ρ and Λ. An efficient evaluation of *T*_2_ is critical in our approach to avoid additional cost relative to the conventional RPA in the dielectric matrix formulation.

We express the amplitudes in terms of the singlet excitation operators^41^
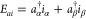
31

The closed-shell direct-ring double excitation amplitudes
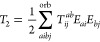
32are the solution of the ring coupled-cluster doubles equation^37^
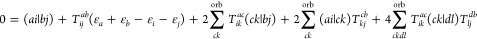
33

Remarkably, the exact solution of eq 33 can be computed in a single shot with the matrices already available in the Cholesky/frequency-integral RPA implementations. Let us denote the matrix of the vovo Coulomb integrals as
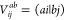
34

As usual, *V* will be used in a Cholesky-decomposed form^4^
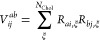
35where *R*_*ai*,ξ_ denotes the Cholesky vectors and *N*_Chol_ is proportional to the orbital basis-set dimension. Additionally, let us represent the orbital energy differences as a diagonal matrix
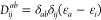
36

With those definitions, the matrix form of the doubles equation is
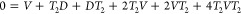
37

The solution of eq 37 assumes the form
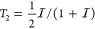
38where the auxiliary matrix
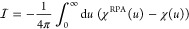
39is determined from the RPA response matrix, χ^RPA^, i.e., the solution of the RPA screening equation at full coupling strength
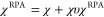
40

The computational form of eq 39 employs the Cholesky decomposition of *V* and is given by
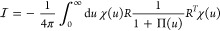
41where χ(*u*) is the closed-shell noninteracting density-response function at frequency *u*
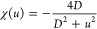
42and Π(*u*) is the auxiliary matrix defined in ref (15):
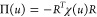
43

In our implementation, the RPA amplitudes are available only in the form of a set of eigenvalues *a*_κ_ and eigenvectors *U*_*ai*,κ_:
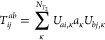
44

The numerical rank of *T*_2_ is a multiple of the number of the Cholesky vectors of *V* and, consequently, scales linearly with the system size. The eigenvalues of *T*_2_ satisfy
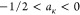
45which prevents division by 0 in the matrix formulas for δρ and Λ (see Section 4 of the Supporting Information). We note that the direct-ring amplitudes *T*_2_ lack the positive part of the eigenvalue spectrum, cf. Figure 1 in ref (42). See Section 1 of the Supporting Information for the proofs of eq 38, the bound on the eigenvalues, and the description of the algorithm for the  numerical evaluation of *T*_2_.

**Figure 1:**
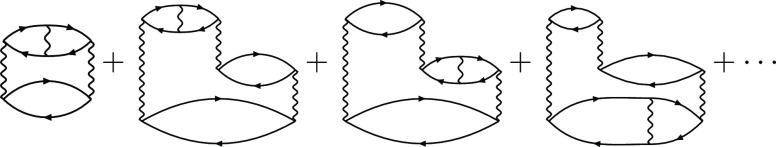
Expansion of the third-order beyond-RPA contribution *E*_c_^2*g*^. Equation 51 accounts for the correct prefactor of the leading diagram, but the higher-order terms are inexact.

### 2.5 CCD Correction

Consider the CCD expectation value of the full normal-ordered Hamiltonian
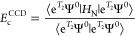
46evaluated with the RPA doubles, *T*_2_, of eq 38. We will derive the CCD correction to the RPA correlation energy in the form of
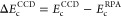
47

The RPA doubles are easy to compute but accurate only through first order. Fortunately, Wigner’s 2*n* + 1 rule^38,43^ applies to eq 46, which means that the first-order *T*_2_ is sufficiently accurate to get the full set of pure-doubles third-order terms. Using ρ and Λ in the quadratic CCD approximation and in the infinite-order ring approximation (Section 4 in the Supporting Information), one can show that *E*_c_^CCD^ expands into
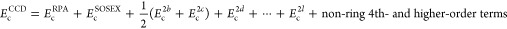
48where the first two terms on the rhs are the direct-ring RPA correlation energy, eq 1, or equivalently in the coupled-cluster formulation^37^
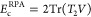
49and the SOSEX correction^24^
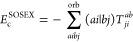
50

The remaining explicit contributions, *E*_c_^2*b*^, *E*_c_^2*c*^, ..., *E*_c_^2*l*^, originate from the CCD cumulant and, together with *E*_c_^SOSEX^, form the complete set of third-order beyond-RPA energy corrections of the pure doubles type. The orbital-level formulas are listed in Table 2. The remainder of eq 48 contains non-ring contractions of *T*_2_ and *V*, where *T*_2_ is at least in the third power. The details of the derivation of eq 48 are described in Section 5 of the Supporting Information.

**Table 2:** Linear and Quadratic Contributions to the Cumulant Part of the CCD Expectation Value of the Hamiltonian^a^

trace contribution	intermediates
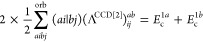	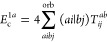
	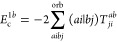
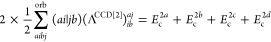	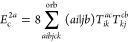
	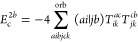
	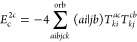
	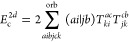
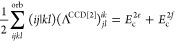	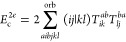
	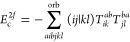
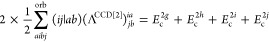	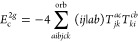
	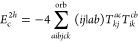
	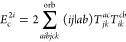
	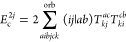
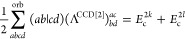	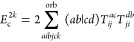
	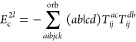

a Numerical prefactors related to the permutational symmetry of Λ_*qs*_^*pr*^ are indicated in the first column in front of each summation symbol. The quadratic CCD approximation of the cumulant matrix is denoted as Λ^CCD[2]^.

In the workable variant of Δ*E*_c_^CCD^, we will retain only a small subset of terms from eq 48 to stay within the cost of a conventional RPA calculation. As proposed by Masur et al. in the context of local coupled-cluster calculations,^44^ the criterion for the selection of the CCD diagrams can be their decay rate in remote subsystems. It has been concluded in ref (44) that the non-ring diagrams analogous to those in Figure 1 are particularly relevant in noncovalent interactions due to the slow 1/*R*^6^ decay. To account for this contribution in the third order, we include the *E*_c_^2*g*^ term
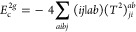
51which also happens to be one of the two least-expensive beyond-RPA terms in eq 48. The other inexpensive term which we include is the SOSEX correction,^24^ which decays fast with *R* but is required to have the complete second order of MBPT. *E*_c_^SOSEX^ is well-established in the literature, but here we apply it in the semicanonical basis, as is the case for all correlation energy components. To summarize,
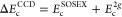
52is the final variant of the doubles correction to be paired with the 1-RDM (singles) correction in the implemented variant of the beyond-RPA approach. The  implementation of eq 52 is described in Section 2.7.

### 2.6 1-RDM Correction

At this point, the proposed approximation of *E*_c_ still misses the contributions from single excitations. A subset of those contributions in the second and third orders is presented in Table 1 as diagrams I, II, III, and IV. To obtain the complete set of third-order single-excitation terms, one should also add eight additional diagrams with two *V* operators and a single perturbation δ*h* (see diagrams 5–12 in Figure 14b of ref (31).). We will focus on diagrams I–IV because those can be accounted for simply by using the mean-field approximation of *E*_c_^1RDM,lin^ and *E*_c_^1RDM,quad^, as follows. Consider the eigenproblem of the full one-electron Hamiltonian
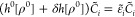
53where the zeroth order 1-RDM, ρ^0^, in square brackets indicates that the Fock operator is computed using the Kohn–Sham density matrix. The mean-field 1-RDM
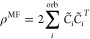
54includes pure δ*h* terms up to infinite order. Now, inserting the mean-field 1-RDM difference
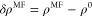
55into eqs 20 and 21 yields the mean-field approximation of *E*_c_^1RDM^
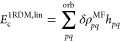
56
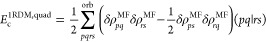
57

As shown in Section 3 of the Supporting Information, eqs 56 and 57 expand into terms I–IV with the exact MBPT prefactors. The mean-field *E*_c_^1RDM,lin^ is exactly equivalent to the Hartree–Fock singles correction of Klimeš et al.^27^Equation 56 also agrees through the third order with the singles correction in the rPT2 method of Ren et al.^23^ In the fourth order, *E*_c_^1RDM,lin^ includes additional pure δ*h* diagrams not accounted for in the rPT2 energy, although this difference appears to be numerically insignificant. Contributions III and IV which originate from *E*_c_^1RDM,quad^ are not accounted for in any of the existing RPA singles corrections.^23,27^ Higher-order correlation effects involving singles can be added by using the coupled-cluster expectation-value techniques with some approximation of *T*_1_, but this is beyond the scope of this work. See Section 3 of the Supporting Information for the details of the MBPT expansion of *E*_c_^1RDM,lin^ and *E*_c_^1RDM,quad^.

### 2.7 Tensor Hypercontraction of Coulomb Integrals

The cost of *E*_c_^SOSEX^ and *E*_c_^2*g*^ in their base form is  but can be reduced to  with low-rank tensor decomposition. The eigen decomposition of eq 44 decouples the orbital pair indices *ai* and *bj* in *T*_*ij*_^*ab*^. The tensor hypercontraction (THC) decomposition^30,30,45–47^ decouples the indices in the Coulomb integral
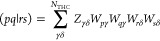
58

The matrix *Z*_γδ_ is the THC grid representation of the Coulomb operator. *W*_*p*γ_ is the vector of the *p*th orbital values on the THC molecular grid.^30,45^ The THC molecular grid size, *N*_THC_, is on the order of a few hundred nodes per atom.^48^ The computational form of *E*_c_^SOSEX^ and *E*_c_^2*g*^ in terms of the amplitude eigenvectors and THC vectors is
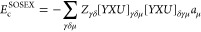
59
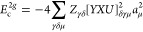
60where [*YXU*]_γδμ_ is an intermediate defined as
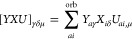
61with *Y*_*a*γ_ and *X*_*i*γ_ representing the *W*_*p*γ_ vectors transformed to the molecular orbital basis
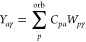
62
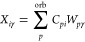
63*C*_*pa*_ and *C*_*pi*_ are the molecular-orbital coefficients of the *a*th virtual orbital and the *i*th occupied orbital, respectively. The matrix elements [*YXU*]_γδμ_ are formed on the fly and discarded immediately after being used. Equations 59 and 60 contain only a single loop over the eigenvector index μ, which means that the eigenvectors of *T*_2_ can be generated, used to update *E*_c_^SOSEX^ and *E*_c_^2*g*^ and immediately discarded to decrease memory usage.

## 3 Numerical Results

The numerical section is designed to assess the differences in noncovalent interaction energies between the proposed approach and rPT2,^23^ the state of the art in the quartic-scaling category. The total energy in the implemented variant of our approach gathers the terms defined in eqs 1, 56, 57, 50, and 51 and the reference mean-field energy *E*^HF^ of eq 5
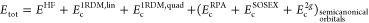
64

The total rPT2 energy is^23^
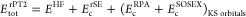
65

The terms enclosed in parentheses are evaluated in the molecular-orbital basis indicated by the lower label. The beyond-RPA MBPT terms accounted for in eq 64 are summarized in Table 1 and in Figure 1. The rPT2 completely lacks third-order diagrams III, IV, V, and VI in Table 1 as well as the diagrams in Figure 1. The renormalized singles energy, *E*_c_^rSE^ in eq 65, is numerically almost indistinguishable from *E*_c_^1RDM,lin^, but formally those terms differ in the fourth order (see Section 3 of the Supporting Information).

### 3.1 Computational Details

The beyond-RPA code was added on top of the RPA program described in refs (15) and (16). All beyond-RPA calculations were preceded by the PBE self-consistent field^32^ on a dense molecular grid with 150 radial and 590 spherical points. The correlation energy components *E*_c_^RPA^, *E*_c_^SOSEX^, and *E*_c_^2*g*^ were obtained within the frozen-core approximation and extrapolated to the basis set limit using the formula of Halkier et al.^50^ (aug-cc-pVTZ → aug-cc-pVQZ). The frequency integration grid applied in eq 38 was the same as the direct RPA grid in refs (15) and (16). The Cholesky decomposition of the Coulomb integrals with the diagonal elements threshold τ = 10^–7^, defined as given in ref (51), was applied both in the SCF step and in the RPA correlation energy calculation. The tensor hypercontraction decomposition of atomic-orbital Coulomb integrals was implemented according to ref (48) with the THC cutoff threshold ε = 10^–3^. The THC decomposition was applied only in the post-SCF part of the energy. A recalculation of the results for CH_4_···H_2_O and CH_4_...(H_2_O)_2_ with a tighter THC threshold ϵ = 10^–5^ changed the individual dimer and trimer interaction energy contributions by less than 10^–5^ kcal/mol. All noncovalent interaction energies were computed with molecular geometries fixed at their interacting cluster values, i.e., without the effect of geometry relaxation. All energies contributing to the *n*-body (nonadditive) interaction energy were computed in the basis set of the corresponding *n*-body cluster.

### 3.2 Interactions of Dimers

We test the interaction energies on a set of small-molecule dimers for which the dispersion-to-electrostatic ratio (disp/elst) ranges from disp/elst = 0.4 for the methanol-methylamine complex up to disp/elst = 4.5 for the ethene–pentane dimer. The disp/elst ratio is defined as in ref (29). The systems are a subset of the S66 × 8 database.^29^ The reference CCSD(T) and CCSD interaction energy data are taken from ref (49).

**Figure 2:**
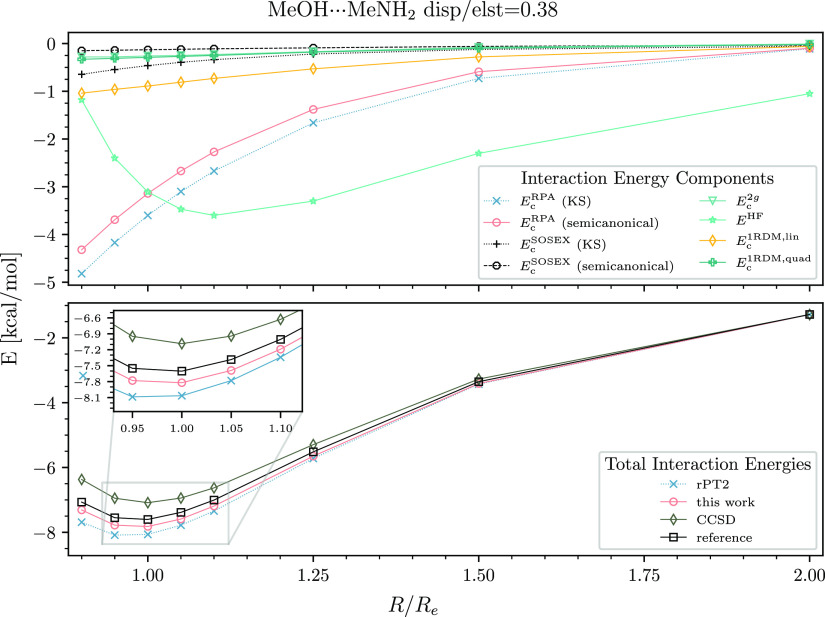
Interaction energy curve of the methanol-methylamine dimer. The upper panel shows the interaction energy contributions corresponding to the single-point energy terms in eqs 64 and 65. The curve denoted as *this work* is the sum of *E*^HF^, *E*_c_^1RDM,lin^, *E*_c_^1RDM,quad^, *E*_c_^RPA^, *E*_c_^SOSEX^, and *E*_c_^2*g*^, applied with the semicanonical orbitals. The rPT2 energy^23^ is the sum of *E*^HF^, *E*_c_^RPA^, *E*_c_^rSE^, and *E*_c_^SOSEX^, applied with the KS orbitals. On the scale of the plot, *E*_c_^rSE^ of ref (23) and *E*_c_^1RDM,lin^ would be indistinguishable. The reference CCSD(T) and CCSD curves are taken from ref (49). The disp/elst ratio is taken from ref (29)

**Figure 3:**
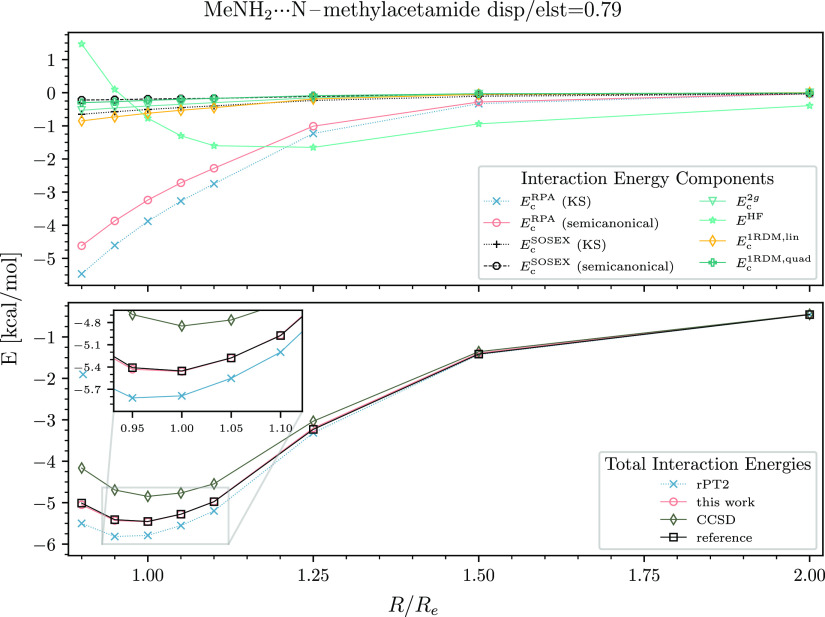
Interaction energy curve of the methylamine-*N*-methylacetamide dimer. See the caption of Figure 2 for details.

**Figure 4:**
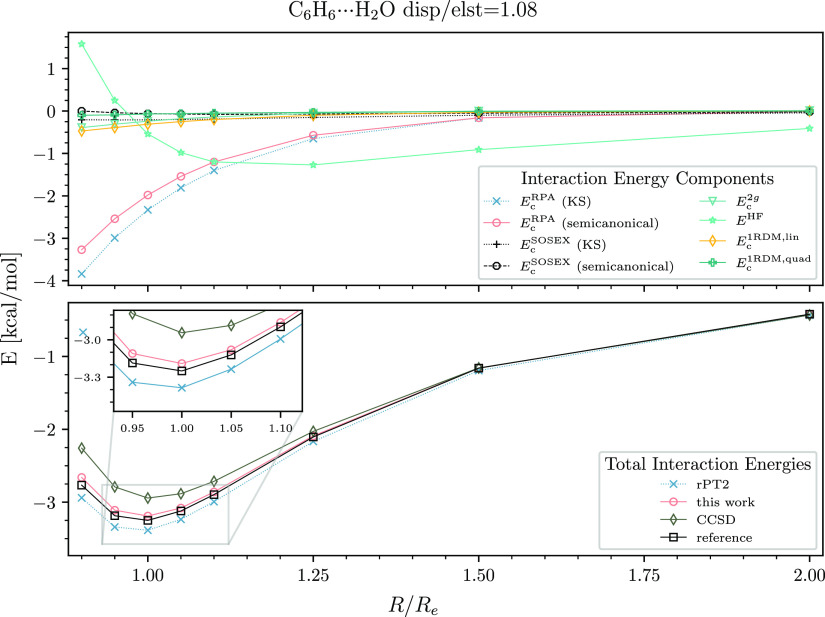
Interaction energy curve of the benzene-water dimer. See the caption of Figure 2 for details.

**Figure 5:**
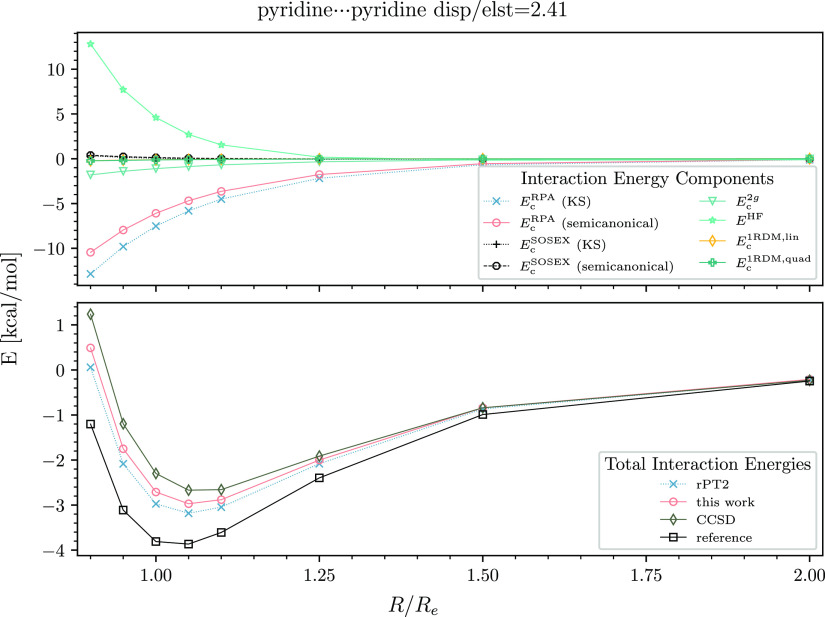
Interaction energy curve of the pyridine dimer. See the caption of Figure 2 for details.

**Figure 6:**
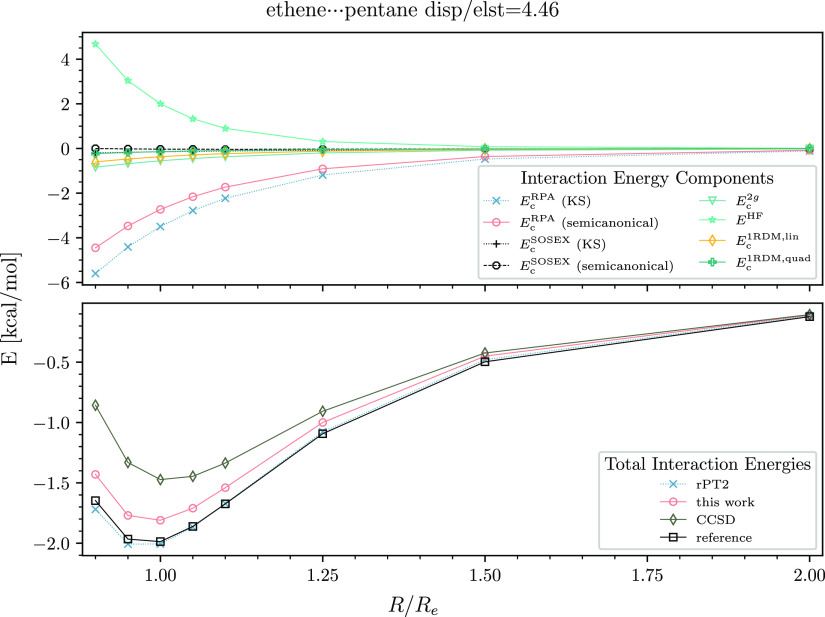
Interaction energy curve of the ethene–pentane dimer. See the caption of Figure 2 for details.

The disp/elst ratio is the key descriptor that determines the behavior of the RPA methods. In the low-dispersion (polar) systems, the errors of our approach are small, ca. 0.2 kcal/mol for methanol-methylamine and only a few hundredths of a kcal/mol for methylamine-*N*-methylacetamide (Figures 2 and 3). In the moderate dispersion case of benzene–water, disp/elst = 1.1, a slight underbinding appears, but the error is still small: 0.1 kcal/mol at the equilibrium distance (Figure 4). However, for typical high-dispersion nonpolar systems, the binding strength is visibly underestimated. In the worst case of pyridine–pyridine, the error is 1.1 kcal/mol at equilibrium (Figure 5). Note that in this case, all approximate methods, including CCSD, behave similarly. This indicates the importance of connected triples in the pyridine dimer. For the dimer with the largest disp/elst ratio, ethene–pentane, the interaction energy at equilibrium is underestimated by 0.2 kcal/mol (Figure 6).

The rPT2 method exhibits a different pattern. The binding strength of polar dimers tends to be excessive: the errors for methanol-methylamine and methylamine-*N*-methylacetamide at equilibrium are 0.5 and 0.3 kcal/mol, respectively. The high-dispersion cases are described better. The error for pyridine–pyridine is 0.8 kcal/mol, which is smaller than the errors in both the CCSD method and our approach. The results for the ethene–pentane dimer match the reference to within a few hundredths of a kcal/mol.

The marked difference in the binding strength between our approach and that of rPT2 is related to the use of the semicanonical basis. As seen in the upper panels of Figures 2–6, the *E*_c_^RPA^ contribution to the interaction energy is always less attractive in the semicanonical basis. For example, for the ethene–pentane dimer at the equilibrium distance, the direct RPA contribution to the interaction energy is −3.5 kcal/mol in the KS basis and only −2.7 kcal/mol in the semicanonical basis. Also the semicanonical *E*_c_^SOSEX^ term is less attractive than its KS-orbital counterpart in polar dimers. At the equilibrium distance of the methanol-methylamine dimer, the KS-orbital *E*_c_^SOSEX^ contributes −0.46 kcal/mol, while its semicanonical counterpart contributes only −0.13 kcal/mol. For nonpolar systems, *E*_c_^SOSEX^ is small regardless of the orbitals.

The contributions of *E*_c_^rSE^ and *E*_c_^1RDM,lin^ would be visually indistinguishable on the scale of the presented plots. Therefore, we draw only *E*_c_^1RDM,lin^ in the upper panels of Figures 2–6. It is clear from our data that *E*_c_^1RDM,lin^ is important for all types of dimers. The quadratic mean-field change term is smaller than *E*_c_^1RDM,lin^ but still significant. *E*_c_^1RDM,quad^ is up to −0.3 kcal/mol for the polar dimers and up to −0.2 kcal/mol for moderate- and high-dispersion systems.

The behavior of the semicanonical RPA correlation energy resembles, to some extent, the well-known underbinding error of RPA on the self-consistent Hartree–Fock reference. However, in the proposed approach, the third-order correction term *E*_c_^2*g*^ compensates for diminished attraction. In the case of the ethene–pentane dimer at the equilibrium distance, *E*_c_^2*g*^ contributes −0.55 kcal/mol, which is one-fourth of the total binding effect. *E*_c_^2*g*^ is comparable in magnitude to *E*_c_^1RDM,lin^ in the moderate- and high-dispersion cases but smaller than *E*_c_^1RDM,lin^ for the polar dimers.

It is clear from our results that only the semicanonical-orbital RPA can accommodate multiple beyond-RPA attractive terms without severe overestimation of the interaction energy. This should be contrasted with the KS-orbital approach, where the interaction energy curve for hydrogen-bonded systems is too deep when both *E*_c_^rSE^ and *E*_c_^SOSEX^ are present, but the simpler *E*_c_^RPA^ + *E*_c_^rSE^ approach is remarkably accurate.^23^

### 3.3 Methane in a Dodecahedral Water Cage

The binding energy of the methane molecule in a dodecahedral water cage is a finite model of the dispersion-dominated interaction in a methane hydrate.^13,52^ It is an interesting example of a noncovalent system where all local DFT approximations fail due to the inability to account for the interaction nonadditivity.^13,16^ In this context, developing low-scaling approaches for the *n*-body interactions, like the method presented in this work, is desirable given their essential role in the incremental energy calculation in molecular crystals.^53,54^ In what follows, the total CH_4_...(H_2_O)_20_ interaction energy
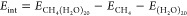
66is decomposed into two-, three-, and four-body contributions
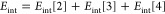
67

The nonadditive interactions of *n* > 4-body clusters are not considered. *E*_int_[2] is the sum of 20 interaction energies of CH_4_...H_2_O dimers
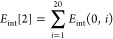
68where the pairwise interaction energy is
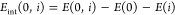
69*E*_int_[3] is the sum of 190 three-body nonadditive energies of CH_4_...(H_2_O)_2_
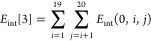
70where the nonadditive energy of a trimer is the total binding energy minus the pairwise contributions
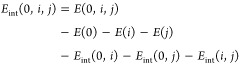
71

Finally, *E*_int_[4] is the sum of 1140 four-body nonadditive energies of CH_4_...(H_2_O)_3_
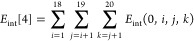
72where the four-body nonadditivity is defined as the tetramer binding energy minus the pairwise energies and three-body nonadditivities
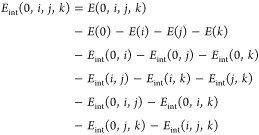
73

The indices *i*, *j*, and *k* in eqs 68–73 correspond to the water molecules; 0 corresponds to the methane molecule.

Let us start by noting that there are large errors in the self-consistent PBE model, which is the base for the tested RPA methods. It is evident from Table 3 that PBE not only fails to capture the pairwise interaction but also estimates the nonadditive three- and four-body effects to be nearly an order of magnitude larger than the reference values. It is thus expected that the errors in the underlying PBE functional will lead to significant corrections for the mean-field change. Indeed, as the cluster size increases from two to three and finally to four molecules, both *E*_c_^1RDM,lin^ and *E*_c_^1RDM,quad^ alternate their signs but do not decay. The absolute value of the *E*_c_^1RDM,lin^ contribution is over 2 times larger than the total three-body nonadditive energy and over 5 times larger than the total four-body nonadditivity. The *E*_c_^1RDM,quad^ contribution, which we found relatively small for the individual dimer interaction–energy curves, is now important. For instance, the three-body *E*_c_^1RDM,quad^ term amounts to 0.85 kcal/mol out of the total three-body contribution of 1.36 kcal/mol.

**Table 3:** Many-Body Expansion of the Binding Energy of CH_4_ in Dodecahedral Water Cage CH_4_(H_2_O)_20_^a^^i^

	dimers^b^	trimers^c^	tetramers^d^	∑
CCSD(T) Reference^e^				
*E*_tot_	–6.31	1.04	0.56	–4.72
*E*^HF^	3.83	–0.27	0.55	4.11
*E*_c_^CCSD^	–8.50	1.11	–0.02	–7.41
*E*_c_^(T)^	–1.64	0.20	0.02	–1.42
This Work^f^ (Semicanonical Orbitals)				
*E*_tot_	–6.45	1.36	0.41	–4.69
*E*^HF^	7.69	–4.35	3.65	6.99
*E*_c_^1RDM,lin^	–2.83	2.95	–2.23	–2.10
*E*_c_^1RDM,quad^	–0.77	0.85	–0.65	–0.57
*E*_c_^RPA^	–8.69	1.37	–0.24	–7.56
*E*_c_^2*g*^	–1.83	0.44	–0.10	–1.49
*E*_c_^SOSEX^	–0.03	0.09	–0.02	0.04
rPT2^g^ (KS Orbitals)				
*E*_tot_	–6.99	0.94	1.00	–5.05
*E*^HF^	7.69	–4.35	3.65	7.00
*E*_c_^rSE^	–2.83	2.95	–2.23	–2.10
*E*_c_^RPA^	–11.56	2.34	–0.61	–9.83
*E*_c_^SOSEX^	–0.29	0.00	0.18	–0.11
DFT(PBE)^h^				
*E*_tot_	–3.88	7.45	–3.46	0.11

a All energies are denoted in kilocalories per mol.

b Sum of 20 interaction energies of CH_4_...H_2_O.

c Sum of 190 three-body nonadditive energies of CH_4_...(H_2_O)_2_.

d Sum of 1140 four-body nonadditive energies of CH_4_...(H_2_O)_3_.

e Taken from ref (16).

f Single-point energy contributions defined in eq 64.

g Renormalized second-order perturbation theory of Ren et al.^23^*E*_c_^rSE^ denotes the renormalized singles energy defined in ref (23).

h Self-consistent DFT calculations with the PBE exchange–correlation model.

i RPA and beyond-RPA energy components are extrapolated (aug-cc-pVTZ → aug-cc-pVQZ) using the method of Halkier et al.^50^

In accordance with the results for the dimer curves, the two-body semicanonical direct-ring contribution to the methane-binding energy is less attractive than that of the KS-orbital counterpart (Table 3). The semicanonical two-body contribution of *E*_c_^RPA^ amounts to −8.7 kcal/mol while that of the KS-orbital counterpart is as large as −11.5 kcal/mol. This is partially corrected by the *E*_c_^2*g*^ term, which contributes −1.8 kcal/mol to the two-body binding energy. The pattern remains the same in clusters with more than two molecules, where the semicanonical direct-ring contribution is again smaller, but *E*_c_^2*g*^ corrects the result. The semicanonical SOSEX contribution is small for all types of clusters. The magnitude of the KS-orbital SOSEX term is larger, but the two-body contribution is nearly canceled by the four-body term, which reduces the total SOSEX contribution to only −0.1 kcal/mol.

After summation over all cluster types, the total methane-binding energy in our approach equals −4.7 kcal/mol, which nearly perfectly matches the reference value. This agreement is the result of a fortuitous cancellation between a slightly too attractive two-body contribution and too repulsive nonadditivity. The rPT2 method overshoots the reference total binding energy by about 0.3 kcal/mol, mainly due to the large overestimation of the two-body contribution. Overall, the dominant trend seen in dispersion-driven dimers holds true for CH_4_···(H_2_O)_20_. While semicanonical orbitals greatly diminish the binding effect of *E*_c_^RPA^, the total energy remains accurate thanks to the beyond-RPA terms *E*_c_^2*g*^ and *E*_c_^1RDM,quad^.

## 4 Conclusions

We have shown a systematic beyond-RPA approach where the correlation energy approximation is derived from expectation-value coupled-cluster theory. The base RPA is the ring part of the coupled-cluster doubles expectation value of the normal-ordered Hamiltonian. The remaining contributions are the beyond-RPA corrections in the form of (i) the coupled-cluster doubles non-ring correction, Δ*E*_c_^CCD^, evaluated with the RPA amplitudes, *T*_2_, (ii) and the singles correction, expressed with the mean-field one-electron reduced density matrix. The doubles part, Δ*E*_c_^CCD^, takes advantage of Wigner’s 2*n* + 1 rule and is potentially accurate through the third order, but in practice we restrict it to only two least expensive terms, *E*_c_^SOSEX^ and *E*_c_^2*g*^. The cost of the resulting correlation energy is noniterative .

The proposed approach includes all beyond-RPA terms of the state-of-the-art rPT2 method, i.e., the linear singles correction and the second-order screened exchange term but supplements those with novel elements: (i) the semicanonical orbital basis, (ii) the non-ring third-order term *E*_c_^2*g*^, (iii) and the quadratic 1-RDM (singles) correction *E*_c_^1RDM,quad^.

The semicanonical basis employed in *E*_c_^RPA^ and *E*_c_^SOSEX^ accounts for the third- and higher-order terms which arise for non-Hartree–Fock Hamiltonians.^18,33,39^ While those contributions are justified from the MBPT perspective, they greatly diminish the binding strength relative to the conventional KS basis. Fortunately, this effect is counteracted by the proposed beyond-RPA terms *E*_c_^2*g*^ and, to a lower extent, *E*_c_^1RDM,quad^. The semicanonical-orbital basis resolves the incompatibility of SOSEX and the singles correction observed by Ren et al. in polar and hydrogen-bonded systems.^23^ The SOSEX correction computed with semicanonical orbitals is small and no longer causes overbinding in this type of systems.

The third-order doubles term, *E*_c_^2*g*^, accounts for a large fraction of the interaction energy in dispersion-dominated dimers, e.g., *E*_c_^2*g*^ contributes nearly one-third of the binding effect in the CH_4_···(H_2_O)_20_ cluster. From the perspective of noncovalent interactions, *E*_c_^2*g*^ is a beyond-RPA contribution of primary importance, with a computational cost that is comparable to the well-established SOSEX correction.

The essential role of the singles correction in post-KS beyond-RPA methods is already recognized in the literature, but we have shown that apart from the usual linear singles correction, the quadratic term, *E*_c_^1RDM,quad^, becomes important in many-body noncovalent systems. This result emphasizes the need to account for the change of the mean-field energy in systems where the underlying DFT exchange–correlation model poorly describes the nonadditive component of intermolecular interactions.

As a general observation, the proposed approach is accurate in polar and mixed-type systems but underbinds high-dispersion complexes. This contrasts with the trend seen in the rPT2 results, where the energies of high-dispersion systems are accurate, while polar dimers are bound too strongly. If considered as an approximation within the proposed framework, the performance of rPT2 can be explained by the presence of systematic error cancellation. The rPT2 method neglects both the attractive term *E*_c_^2*g*^ and the repulsive contributions which arise in MBPT for the non-Hartree–Fock reference.

Finally, we note that the presented scheme allows for the inclusion of further beyond-RPA terms from Table 2, provided that an efficient implementation is developed. Work is currently underway to include additional exchange terms and to survey various third-order contributions to a broader range of molecular properties.

## Supporting Information Available

Additional derivations and spreadsheets with raw numerical data (XLSX)
Many-body expansion of the binding energy of CH_4_ in dodecahedral water cage (XLSX)
RPA double-excitation amplitudes, semicanonical basis, MBPT analysis of E_c_^1RDM^, coupled-cluster expectation-value theory, beyond-RPA terms in the expectation value of *H*, and adiabatic connection (PDF)
