# Supplementary material for: Post-Kohn–Sham Random-Phase Approximation and Correction Terms in the Expectation-Value Coupled-Cluster Formulation
Source: J Chem Theory Comput. 2023 Sep 29;19(19):6619–31. doi: 10.1021/acs.jctc.3c00496 (PMC10569055; doi:10.1021/acs.jctc.3c00496)
Supplement: Supplementary file 3 — ct3c00496_si_003.pdf [file ct3c00496_si_003.pdf]

Supporting Information: Post-Kohn-Sham random-phase approximation and correction terms in the expectation-value coupled-cluster formulation

Dominik Cieśliński,† Aleksandra M. Tucholska,‡,† and Marcin Modrzejewski*,†

†*Faculty of Chemistry, University of Warsaw, 02-093 Warsaw, Pasteura 1, Poland*

‡*Institute of Physics, Łódź University of Technology, Wólczańska 219, 90-924 Łódź, Poland*

E-mail: m.m.modrzejewski@gmail.com

Contents

1 RPA double excitation amplitudes **S-2**

1.1 Derivation of the matrix formula for T_2 . . . . . . S-2

1.2 Bound on the eigenvalues of T_2 . . . . . . S-4

1.3 Randomized eigendecomposition of T_2 . . . . . . S-5

2 Semicanonical basis **S-6**

3 MBPT analysis of E_c^{1RDM} **S-9**

4 Coupled-cluster expectation-value theory **S-11**

4.1 General expressions S-11

4.2 CCSD reduced-density matrix elements S-13

4.3 RPA auxiliary amplitudes S-15
4.4 RPA+X approximation of the coupled-cluster doubles 1-RDM S-15
4.5 RPA cumulant matrix S-17

5 Beyond-RPA terms in the expectation value of H **S-17**

6 Adiabatic connection **S-21**

References **S-24**

1 RPA double excitation amplitudes

1.1 Derivation of the matrix formula for T_2

We will prove that the matrix formula

$$T_2 = \frac{1}{2}\mathcal{I}/(1+\mathcal{I}) \tag{1}$$

where

$$\mathcal{I} = -\frac{1}{4\pi}\int_0^\infty \mathrm{d}u \left(\chi^{\mathrm{RPA}}(u) - \chi(u)\right) = -\frac{1}{4\pi}\int_0^\infty \mathrm{d}u\, \chi(u) R \frac{1}{1+\Pi(u)} R^T \chi(u) \tag{2}$$

solves the closed-shell RPA doubles equation[S1]

$$0 = (ai|bj) + T_{ij}^{ab}(\epsilon_a + \epsilon_b - \epsilon_i - \epsilon_j) + 2\sum_{ck}^{\mathrm{orb}} T_{ik}^{ac}(ck|bj) + 2\sum_{ck}^{\mathrm{orb}} (ai|ck) T_{kj}^{cb} + 4\sum_{ckdl}^{\mathrm{orb}} T_{ik}^{ac}(ck|dl) T_{lj}^{db} \tag{3}$$

Let us start by casting Eq. 3 in the matrix form

$$0 = V + T_2 D + D T_2 + 2T_2 V + 2V T_2 + 4T_2 V T_2 \tag{4}$$

Inserting Eq. 1 into Eq. 4 and multiplying both sides from left and right by $1+\mathcal{I}$ yields

$$0 = V + 2\mathcal{I}V + 2V\mathcal{I} + 4\mathcal{I}V\mathcal{I} + \frac{1}{2}\mathcal{I}D + \frac{1}{2}D\mathcal{I} + \mathcal{I}D\mathcal{I} \tag{5}$$

We will now perform analytic integration over frequencies in $\mathcal{I}$. In analogy with the derivation of the frequency-integral form of the RPA correlation energy in Ref. S2, we define a positive definite matrix

$$\mathcal{J} = D^2 + 4D^{1/2}RR^TD^{1/2} \tag{6}$$

and apply the Woodbury formula for the inverse of a matrix after a low-rank update[S3]

$$\frac{1}{\mathcal{J}+u^2} + \frac{1}{4}D^{-1}\chi(u) = -\frac{1}{4}D^{-1/2}\chi(u)R\frac{1}{1+\Pi(u)}R^T\chi(u)D^{-1/2} \tag{7}$$

The above identity can be directly checked by multiplication of both sides of Eq. 7 by $\mathcal{J}+u^2$ and a repeated application of $[\chi(u), D] = 0$. The rhs of Eq. 7 is the integrand appearing in $\mathcal{I}$. Integrating both sides of Eq. 7 over frequencies and using the definite integral[S4]

$$\int_0^\infty \frac{1}{\mathcal{J}+u^2}\,\mathrm{d}u = \frac{\pi}{2\sqrt{\mathcal{J}}} \tag{8}$$

yields an analytically integrated form of the auxiliary matrix

$$\mathcal{I} = \mathcal{A} - \frac{1}{2} \tag{9}$$

where

$$\mathcal{A} = \frac{1}{2}D^{1/2}\frac{1}{\sqrt{\mathcal{J}}}D^{1/2} \tag{10}$$

Substitution of Eq. 9 into Eq. 5 yields

$$\frac{1}{4}D = \mathcal{A}D\mathcal{A} + 4\mathcal{A}V\mathcal{A} \tag{11}$$

Finally, the rhs of Eq. 11 can be rearranged into

$$
\begin{aligned}
\mathcal{A}D\mathcal{A} + 4\mathcal{A}V\mathcal{A} &= \mathcal{A}(D + 4V)\mathcal{A} \\
&= \frac{1}{4}D^{1/2}\mathcal{J}^{-1/2}(D^2 + 4D^{1/2}RR^TD^{1/2})\mathcal{J}^{-1/2}D^{1/2} \\
&= \frac{1}{4}D^{1/2}\mathcal{J}^{-1/2}\mathcal{J}\mathcal{J}^{-1/2}D^{1/2} = \frac{1}{4}D
\end{aligned}
\tag{12}
$$

Thereby, Eq. 5 is satisfied and T_2 of Eq. 1 solves the RPA doubles equation.

1.2 Bound on the eigenvalues of T_2

We will prove that all eigenvalues a_κ of T_2 given by Eq. 1 reside within the interval

$$-1/2 < a_\kappa < 0 \tag{13}$$

Let us start by considering the eigenvalues and eigenvectors of $\mathcal{I}$

$$\mathcal{I}U_\kappa = b_\kappa U_\kappa \tag{14}$$

Multiplying Eq. 5 from left and right by U_κ yields

$$0 = U_\kappa^T V U_\kappa + (b_\kappa^2 + b_\kappa)(U_\kappa^T D U_\kappa + 4U_\kappa^T V U_\kappa) \tag{15}$$

Since matrices V and D are positive definite, Eq. 15 can only be satisfied if

$$(b_\kappa^2 + b_\kappa) < 0 \tag{16}$$

which holds if and only if $-1 < b_\kappa < 0$. At the same time, $b_\kappa > -1/2$ is satisfied because $\mathcal{I}$ can be rewritten as Eq. 9 and $\mathcal{A}$ is positive definite. Both conditions considered jointly result in

$$-1/2 < b_\kappa < 0 \tag{17}$$

Taking into account inequality 17 and

$$a_\kappa = \frac{1}{2}\frac{b_\kappa}{1+b_\kappa} \tag{18}$$

it follows that the eigenvalues of T_2 satisfy $-1/2 < a_\kappa < 0$, which ends the proof.

1.3 Randomized eigendecomposition of T_2

The numerical rank of the double-excitation amplitudes matrix has been observed to increase only linearly with the system size.[S5–S7] This creates an opportunity for an efficient and accurate diagonalization without applying conventional algorithms to the full matrix. The small subspace of the physically-relevant eigenvectors of T_2 can be found by random sampling and then a conventional diagonalization algorithm can be applied within that subspace.[S8] At the RPA level, the eigendecomposition of T_2,

$$T_{ij}^{ab} = \sum_{\kappa}^{N_{T_2}} U_{ai,\kappa} a_\kappa U_{bj,\kappa} \tag{19}$$

is computed in $\mathcal{O}(N^4)$ arithmetic operations by the projection of Eq. 1 onto a set of N_{guess} random vectors, where N_{guess} is proportional to the system size.[S9] The details of our implementation are as follows. N_{T_2} is the number of non-negligible eigenvalues controlled by the cutoff threshold ϵ (Algorithm 1). The vectors U_κ are obtained as the eigenvectors of $\mathcal{I}$. The randomized eigendecomposition of $\mathcal{I}$ starts with computing the action of $\mathcal{I}$ on the initial set of N_{guess} columns of independent random numbers from the uniform distribution

$$\mathcal{I}\Omega = \Big(-\frac{1}{4\pi} \sum_{g}^{N_{\text{freq}}} w_g \chi(u_g) R \frac{1}{1+\Pi(u_g)} R^T \chi(u_g) \Big) \Omega \tag{20}$$

where the numerical integration over frequencies employs the points and weights defined in Refs. S10 and S11. The evaluation of the matrix product in Eq. 20 is the bottleneck of the T_2 calculation. To reduce the prefactor, the matrix $\Pi(u)$ is represented in a subspace spanned by

Algorithm 1 Randomized eigendecomposition of T_2.

threshold for discarding redundant vectors: $\epsilon \leftarrow 10^{-10}$
number of random guess vectors: $N_{\text{guess}} \leftarrow 3 \times N_\Pi$
$\Omega'_{ai,\kappa} \leftarrow N_{\text{virt}} N_{\text{occ}} \times N_{\text{guess}}$ independent random numbers from the uniform distribution centered at zero
$\Omega \leftarrow$ normalized columns of Ω'
$Q' \leftarrow \mathcal{I}\Omega$
$Q \leftarrow$ normalized columns of Q'
$G \leftarrow$ the Gram matrix $Q^T Q$
$W'_\kappa, b'_\kappa \leftarrow$ eigenvectors and eigenvalues of G
$U'_{ai,\kappa} \leftarrow \frac{1}{\sqrt{b'_\kappa}} \sum_\mu^{N_{\text{guess}}} Q_{ai,\mu} W'_{\mu\kappa}$ for $\kappa :\ b'_\kappa > \epsilon$
$P \leftarrow U'^T \mathcal{I} U'$
$W_\kappa, b_\kappa \leftarrow$ eigenvectors and eigenvalues of P
eigenvectors of T_2: $U_{ai,\kappa} \leftarrow \sum_\mu^{N_{T_2}} U'_{ai,\mu} W_{\mu\kappa}$
eigenvalues of T_2: $a_\kappa \leftarrow \frac{1}{2} \frac{b_\kappa}{1+b_\kappa}$

N_Π dominant eigenvectors of Π at the lowest frequency of the integration grid, as described in Ref. S10. N_Π is usually significantly smaller than the full dimension of $\Pi(u)$, i.e., the number of the Cholesky vectors. The number of random guess vectors, N_{guess}, should include some level of oversampling to ensure that all significant eigenvectors are included after the initial subspace iteration. In our numerical tests, we have found that setting $N_{\text{guess}} = 3 \times N_\Pi$ is sufficient to reduce the eigendecomposition error below the level of errors from other sources, e.g., the numerical integration over frequencies.

Finally, we note that the linear dependence of N_{T_2} on the system size follows directly from Eq. 20. There is at most $N_\Pi N_{\text{freq}}$ nonzero eigenvalues of $\mathcal{I}$. The number of frequency points N_{freq} depends weakly on the system size and N_Π is at most the number of the Cholesky vectors of V, which increases linearly with the system size. Thus, the numerical rank of N_{T_2} also increases linearly.

2 Semicanonical basis

The occupied and virtual semicanonical orbitals are the eigenvectors of the occupied-occupied and virtual-virtual blocks of the non-selfconsistent Fock hamiltonian computed from the

KS orbitals. We will demonstrate that using the semicanonical orbital basis in the RPA correlation energy formula accounts for an infinite series of MBPT diagrams which appear for non-Hartree-Fock reference hamiltonians. In what follows, we apply a modified variant of the technique described in Appendix C of Ref. S12.

Consider the second-order contribution to the RPA correlation energy, denoted as $E_c^{\mathrm{RPA},(2)}$. We will prove that $E_c^{\mathrm{RPA},(2)}$ evaluated using the semicanonical orbitals and orbital energies accounts for the following class of MBPT diagrams with the one-particle perturbation $\delta h_{pq}^{\mathrm{KS}} = h_{pq} - \delta_{pq}\epsilon_p^{\mathrm{KS}}$:[S13]

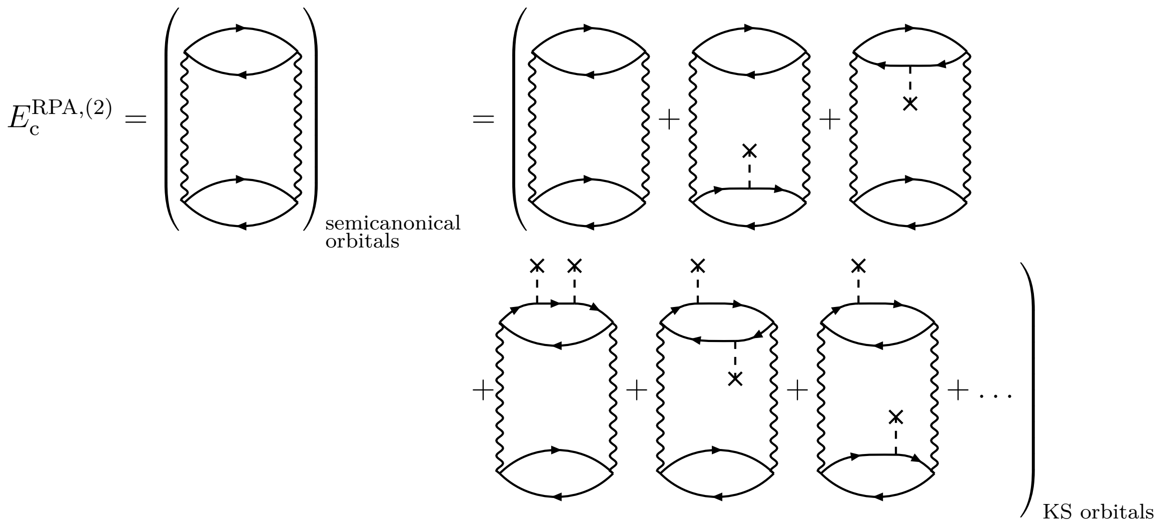

(21)

Using the matrix notation specified in Table S1 and the KS-orbital basis, the sum of diagrams on the rhs of Eq. 21 can be written down as

$$\begin{aligned} E_c^{\mathrm{RPA},(2)} &= 2\boldsymbol{V}^T\boldsymbol{D}^{-1}\boldsymbol{V} + 2\boldsymbol{V}^T\boldsymbol{D}^{-1}\Xi\boldsymbol{D}^{-1}\boldsymbol{V} + 2\boldsymbol{V}^T\boldsymbol{D}^{-1}(\Xi\boldsymbol{D}^{-1})^2\boldsymbol{V} + \dots \\ &= 2\boldsymbol{V}^T(\boldsymbol{D} - \Xi)^{-1}\boldsymbol{V} \end{aligned} \tag{22}$$

The common prefactor of 2 in Eq. 22 follows from the rules of translating MBPT diagrams into closed-shell algebraic formulas:[S14,S15]

$$\text{total prefactor} = \underbrace{(\text{spin summation})}_{4} \times \underbrace{(\text{symmetry factor})}_{1/2} \times \underbrace{(\text{phase factor})}_{\text{accounted for in } \Xi}$$

Table S1: Diagrams contributing to $\mathcal{E}^{(2)}$ and the corresponding matrix formulas.

diagram → matrix notation	matrix elements
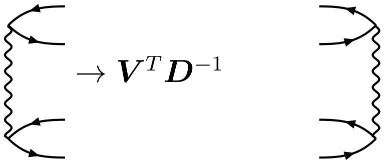 $\rightarrow \boldsymbol{V}^T\boldsymbol{D}^{-1}$ $\rightarrow \boldsymbol{V}$	$\boldsymbol{V}_{aibj} = V_{ij}^{ab}$<br>$\boldsymbol{D}_{aibj,ckdl} = \delta_{ac}\delta_{ik}\delta_{bd}\delta_{jl}\Big(\epsilon_i^{\mathrm{KS}} + \epsilon_j^{\mathrm{KS}} - \epsilon_a^{\mathrm{KS}} - \epsilon_b^{\mathrm{KS}}\Big)$
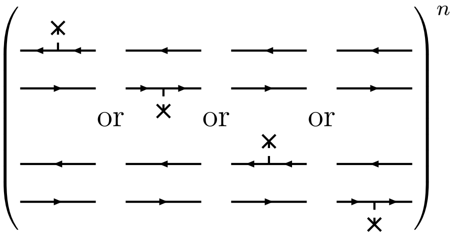 $\rightarrow (\Xi\boldsymbol{D}^{-1})^n$	$\Xi_{aibj,ckdl} = (\delta h^{\mathrm{KS}})_{ac}\delta_{ik}\delta_{bd}\delta_{jl} - \delta_{ac}(\delta h^{\mathrm{KS}})_{ik}\delta_{bd}\delta_{jl} + \delta_{ac}\delta_{ik}(\delta h^{\mathrm{KS}})_{bd}\delta_{jl} - \delta_{ac}\delta_{ik}\delta_{bd}(\delta h^{\mathrm{KS}})_{jl}$<br>$\delta h_{pq}^{\mathrm{KS}} = h_{pq} - \delta_{pq}\epsilon_p^{\mathrm{KS}}$

For the lowest-order diagram, the symmetry factor of 1/2 is the reciprocal value of the order of the group of automorphisms.[S14] For the remaining terms, the order of the group of automorphisms is 1, but the symmetry factor is still 1/2 to prevent double counting of unique diagrams for $(\Xi\boldsymbol{D}^{-1})^n$ with $n \geq 1$. The phase factor depends on the number of hole lines and is accounted for in the definition of Ξ.

The denominator in Eq. 22 can be simplified to the form of

$$(\boldsymbol{D} - \Xi)_{aibj,ckdl} = -h_{ac}\delta_{ik}\delta_{bd}\delta_{jl} + \delta_{ac}h_{ik}\delta_{bd}\delta_{jl} - \delta_{ac}\delta_{ik}h_{bd}\delta_{jl} + \delta_{ac}\delta_{ik}\delta_{bd}h_{jl} \tag{23}$$

Let us transform the matrices in Eqs. 22 and 23 to the semicanonical orbital basis. The semicanonical orbitals will be indicated by the prime symbol. The semicanonical transformation coefficients, $C'_{aa'}$ and $C'_{ii'}$, are the eigenvectors of the occupied-occupied and virtual-virtual blocks of the Fock matrix h

$$\sum_i^{\mathrm{orb}} h_{ji}C'_{ii'} = \epsilon_{i'}C'_{ji'} \tag{24}$$

$$\sum_a^{\mathrm{orb}} h_{ba}C'_{aa'} = \epsilon_{a'}C'_{ba'} \tag{25}$$

The transformation of $\boldsymbol{D} - \Xi$ yields the energy denominator matrix, $\boldsymbol{D}'$, expressed with the

semicanonical orbital energies

$$
\begin{aligned}
\sum_{aibjckdl} C'_{aa'} C'_{ii'} C'_{bb'} C'_{jj'} (\boldsymbol{D} - \boldsymbol{\Xi})_{aibj,ckdl} C'_{cc'} C'_{kk'} C'_{dd'} C'_{ll'} \\
= -(h)_{a'c'} \delta_{i'k'} \delta_{b'd'} \delta_{j'l'} + \delta_{a'c'} (h)_{i'k'} \delta_{b'd'} \delta_{j'l'} - \delta_{a'c'} \delta_{i'k'} (h)_{b'd'} \delta_{j'l'} + \delta_{a'c'} \delta_{i'k'} \delta_{b'd'} (h)_{j'l'} \\
= \delta_{a'c'} \delta_{i'k'} \delta_{b'd'} \delta_{j'l'} (\epsilon'_{i'} + \epsilon'_{j'} - \epsilon'_{a'} - \epsilon'_{b'}) = \boldsymbol{D}'_{a'i'b'j',c'k'd'l'}
\end{aligned} \tag{26}
$$

Finally, using Eq. 26 and the transformed Coulomb integrals matrix, $\boldsymbol{V}'$, we obtain

$$
E_{\mathrm{c}}^{\mathrm{RPA},(2)} = 2\boldsymbol{V}^T (\boldsymbol{D} - \boldsymbol{\Xi})^{-1} \boldsymbol{V} = 2\boldsymbol{V}'^T \boldsymbol{D}'^{-1} \boldsymbol{V}' = \tag{27}
$$

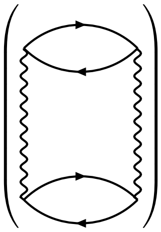

semicanonical

Therefore, the lowest-order contribution to $E_{\mathrm{c}}^{\mathrm{RPA}}$ evaluated in the semicanonical orbital basis accounts for an infinite sum of MBPT diagrams depicted in Eq. 21.

3 MBPT analysis of $E_{\mathrm{c}}^{\mathrm{1RDM}}$

The 1-RDM terms in the correlation energy, $E_{\mathrm{c}}^{\mathrm{1RDM,lin}}$ and $E_{\mathrm{c}}^{\mathrm{1RDM,quad}}$, account for the effect of single excitations from the reference non-Hartree-Fock determinant. At the mean-field level used throughout this work, those contributions are determined using the idempotent density matrix, ρ^{MF}, built from the occupied eigenstates of the mean-field hamiltonian

$$
h = h^0 + \lambda \delta h \tag{28}
$$

at coupling strength $\lambda = 1$. The GMBPT partitioning of the one-electron hamiltonian is assumed.[S16,S17] We will expand $E_{\mathrm{c}}^{\mathrm{1RDM,lin}}$ and $E_{\mathrm{c}}^{\mathrm{1RDM,quad}}$ with respect to the one-electron perturbation, δh, in order to compare the proposed model against the existing approaches of Ren et al.[S12] and Klimes et al.[S18]. The expansion of the mean-field 1-RDM follows from

the commutation relation

$$[h, \rho^{\mathrm{MF}}] = 0 \tag{29}$$

and the idempotency condition

$$\rho^{\mathrm{MF}}\rho^{\mathrm{MF}} = 2\rho^{\mathrm{MF}} \tag{30}$$

Comparing the power-series coefficients on both sides of Eqs. 29 and 30 yields

$$\delta\rho_{ai}^{\mathrm{MF}} = \lambda\frac{2\delta h_{ai}}{\epsilon_i - \epsilon_a} - \lambda^3\sum_{bj}^{\mathrm{orb}}\frac{(\epsilon_i-\epsilon_a)+(\epsilon_i-\epsilon_b)+(\epsilon_j-\epsilon_a)+(\epsilon_j-\epsilon_b)}{(\epsilon_i-\epsilon_a)(\epsilon_i-\epsilon_b)(\epsilon_j-\epsilon_a)(\epsilon_j-\epsilon_b)}\delta h_{bi}\delta h_{bj}\delta h_{aj} + \mathcal{O}(\lambda^5) \tag{31}$$

$$\delta\rho_{ij}^{\mathrm{MF}} = -\lambda^2\sum_{a}^{\mathrm{orb}}\frac{2\delta h_{ia}\delta h_{aj}}{(\epsilon_i-\epsilon_a)(\epsilon_j-\epsilon_a)} + \mathcal{O}(\lambda^4) \tag{32}$$

$$\delta\rho_{ab}^{\mathrm{MF}} = \lambda^2\sum_{i}^{\mathrm{orb}}\frac{2\delta h_{ai}\delta h_{ib}}{(\epsilon_i-\epsilon_a)(\epsilon_i-\epsilon_b)} + \mathcal{O}(\lambda^4) \tag{33}$$

The contributions from Eqs. 31-33 as well as additional fourth-order contributions to the diagonal matrix elements $\delta\rho_{ii}$ and $\delta\rho_{aa}$ lead to the fourth-order expansion of $E_{\mathrm{c}}^{\mathrm{1RDM,lin}}$ and the third-order expansion of $E_{\mathrm{c}}^{\mathrm{1RDM,quad}}$:

$$\begin{aligned} E_{\mathrm{c}}^{\mathrm{1RDM,lin}}[\delta\rho^{\mathrm{MF}}] &= \sum_{pq}^{\mathrm{orb}}\delta\rho_{pq}^{\mathrm{MF}}(h_{pq}^{0}+\lambda\delta h_{pq}) \\ &= 2\lambda^2\sum_{ai}^{\mathrm{orb}}\frac{\delta h_{ai}\delta h_{ia}}{\epsilon_i-\epsilon_a} - 2\lambda^4\sum_{aibj}^{\mathrm{orb}}\frac{\delta h_{ai}\delta h_{bi}\delta h_{aj}\delta h_{bj}}{(\epsilon_i-\epsilon_b)(\epsilon_j-\epsilon_a)(\epsilon_j-\epsilon_b)} + \mathcal{O}(\lambda^6) \end{aligned} \tag{34}$$

$$\begin{aligned} E_{\mathrm{c}}^{\mathrm{1RDM,quad}}[\delta\rho^{\mathrm{MF}}] &= \frac{1}{2}\sum_{pqrs}^{\mathrm{orb}}\left(\delta\rho_{pq}^{\mathrm{MF}}\delta\rho_{rs}^{\mathrm{MF}} - \frac{1}{2}\delta\rho_{ps}^{\mathrm{MF}}\delta\rho_{rq}^{\mathrm{MF}}\right)(pq|rs) \\ &= 8\lambda^3\sum_{aibj}^{\mathrm{orb}}\frac{\delta h_{ai}\delta h_{bj}(ai|bj)}{(\epsilon_i-\epsilon_a)(\epsilon_j-\epsilon_b)} - 2\lambda^3\sum_{aibj}^{\mathrm{orb}}\frac{\delta h_{ai}\delta h_{bj}(aj|bi)}{(\epsilon_i-\epsilon_a)(\epsilon_j-\epsilon_b)} \\ &- 2\lambda^3\sum_{aibj}^{\mathrm{orb}}\frac{\delta h_{ai}\delta h_{bj}(ab|ij)}{(\epsilon_i-\epsilon_a)(\epsilon_j-\epsilon_b)} + \mathcal{O}(\lambda^4) \end{aligned} \tag{35}$$

The terms shown in Eqs. 34 and 35 have the exact prefactors corresponding to the following second-, third-, and fourth-order MBPT diagrams

$E_c^{1RDM,lin}[\delta\rho^{MF}] =$

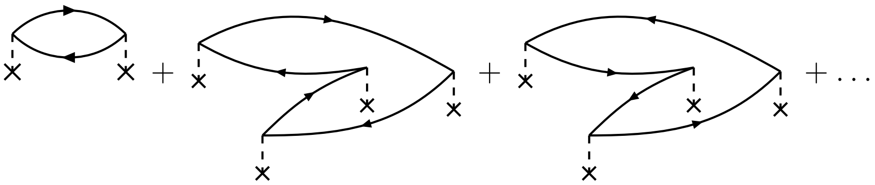

$E_c^{1RDM,quad}[\delta\rho^{MF}] =$

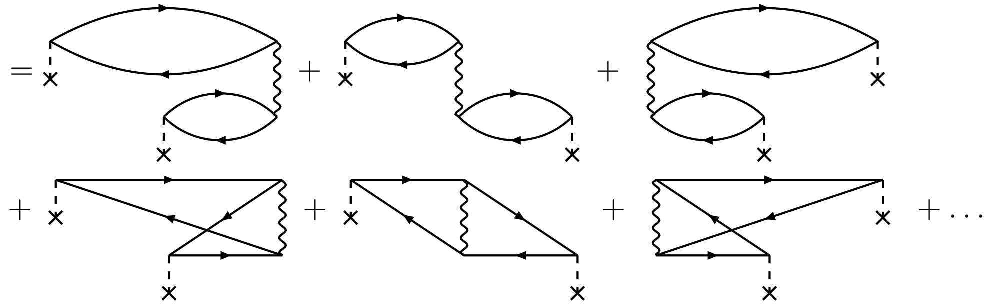

The renormalized singles correction included in the rPT2 method[S12] comprises only the first term in Eq. 34; thus, it lacks the fourth- and higher-order terms in $E_c^{1RDM,lin}$ and the entire $E_c^{1RDM,quad}$ contribution. The renormalized singles correction of Klimes et al. in the mean-field variant, Eq. 32 in Ref. S18, is exactly equivalent to $E_c^{1RDM,lin}[\rho^{MF}]$.

4 Coupled-cluster expectation-value theory

4.1 General expressions

Working expressions for the reduced density matrices follow from the expansion in terms of T of the expectation value in the coupled-cluster formalism. For an arbitrary operator X, the expectation value

$$\frac{\langle\Psi| X |\Psi\rangle}{\langle\Psi|\Psi\rangle} = \frac{\left\langle e^T\Psi^0\right| X \left|e^T\Psi^0\right\rangle}{\langle e^T\Psi^0|e^T\Psi^0\rangle} \tag{36}$$

can be expanded as a commutator series[S19]

$$\frac{\langle\Psi| X |\Psi\rangle}{\langle\Psi|\Psi\rangle} = \left\langle\Psi^0\right| e^{S^\dagger} e^{-T} X e^T e^{-S^\dagger} \left|\Psi^0\right\rangle = \sum_{\mu=0}\sum_{\nu=0} \frac{(-1)^\nu}{\mu!\nu!} \left\langle\Psi^0\right| [[X,T]_\mu, S^\dagger]_\nu \left|\Psi^0\right\rangle \tag{37}$$

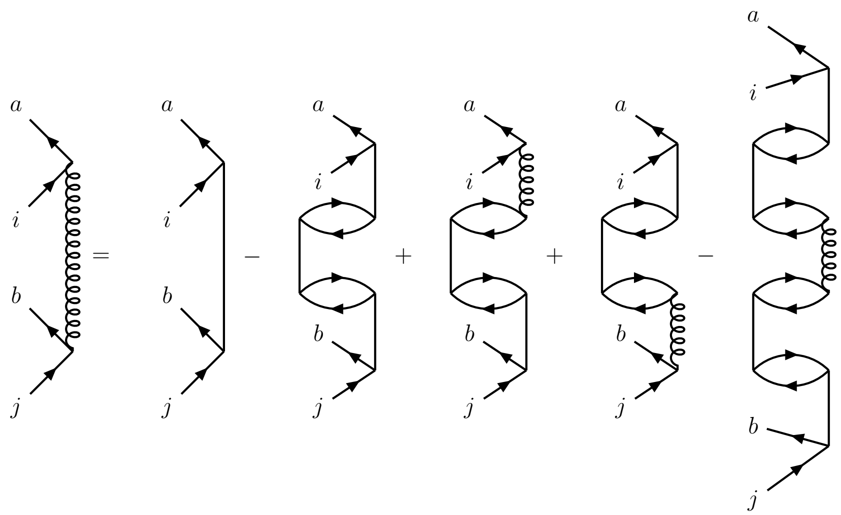

Figure S1: Diagrammatic representation of the S_2 auxiliary amplitude equation in the direct-ring approximation. The operators T_2 and S_2 are represented by the solid and curly lines, respectively.

where the auxiliary excitation operator $S = S_1 + S_2 + \ldots$ is defined by the action on the reference state

$$e^S \left|\Psi^0\right\rangle = \frac{e^{T^\dagger} e^T \left|\Psi^0\right\rangle}{\left\langle\Psi^0\right| e^{T^\dagger} e^T \left|\Psi^0\right\rangle} \tag{38}$$

The operator S technique in the variational coupled-cluster formulation has been introduced by Arponen.[S20] However, later work of Jeziorski and Moszyński have shown that the auxiliary amplitudes can be obtained more conveniently from the commutator expansion[S19]

$$S_n = T_n - \frac{1}{n}\mathcal{P}_n\left(\sum_{\mu=1}\sum_{m=1}\frac{m}{\mu!}[(T_m)^\dagger, T]_\mu + \sum_{\mu=1}\sum_{\nu=0}\sum_{m=1}\frac{m}{\mu!\nu!}[[S_m, T^\dagger]_\mu, T]_\nu\right) \tag{39}$$

The nested commutator in Eqs. 37 and 39 is defined by the recurrence

$$[A, B]_0 = A \tag{40}$$

$$[A, B]_{\mu+1} = [[A, B]_\mu, B] \tag{41}$$

The operator $\mathcal{P}_n(A)$ projects onto the n-tuply excited part of A, e.g., $\mathcal{P}_2(T) = T_2$. The expectation-value formalism with the auxiliary amplitudes allows expressing reduced density

matrices needed for the correlation energy as explicit functions of the RPA amplitudes, avoiding the need to solve the response equation.[S21]

4.2 CCSD reduced-density matrix elements

The reference level of approximation for ρ and Λ is obtained by applying the scheme described in Section 4.1 with two assumptions: (i) the cluster operator T and the auxiliary operator S include only single and double excitations; (ii) the formulas derived from Eq. 37 are expanded in T and truncated after the quadratic terms. The quadratic singles and doubles approximation is referred to as CCSD[2]. The quadratic doubles approximation, CCD[2], is obtained by removing all terms involving T_1 from the CCSD[2] expressions.

The closed-shell 1-electron density matrix in the coupled-cluster parametrization is given as the expectation value

$$\rho_{pq} = \left\langle \Psi^0 \middle| e^{S^\dagger} e^{-T} E_{pq} e^{T} e^{-S^\dagger} \middle| \Psi^0 \right\rangle \tag{42}$$

After substitution of $T = T_1 + T_2$ and expansion of S in terms of T, the orbital-level formulas for the virtual-occupied, occupied-occupied, and virtual-virtual blocks of $\rho^{\text{CCSD[2]}}$ are as follows:

$$\delta\rho_{ai}^{\text{CCSD[2]}} = 2T_{ai} + \sum_{bj}^{\text{orb}} \left(4T_{bj}T_{ji}^{ba} - 2T_{bj}T_{ij}^{ba} \right) \tag{43}$$

$$\delta\rho_{ij}^{\text{CCSD[2]}} = -2\sum_{a}^{\text{orb}} T_{ai}T_{aj} + \sum_{abk}^{\text{orb}} \left(-4T_{ik}^{ab}T_{kj}^{ba} + 2T_{ik}^{ab}T_{jk}^{ba} \right) \tag{44}$$

$$\delta\rho_{ab}^{\text{CCSD[2]}} = 2\sum_{i}^{\text{orb}} T_{ai}T_{bi} + \sum_{cij}^{\text{orb}} \left(4T_{ij}^{ac}T_{ji}^{cb} - 2T_{ij}^{ac}T_{ij}^{cb} \right) \tag{45}$$

The cumulant matrix in the coupled-cluster parametrization has been introduced by Korona

in Refs. S22 and S23. The closed-shell cumulant matrix element[S24]

$$\Lambda^{pr}_{qs} = \Gamma^{pr}_{qs} - \left(\rho_{pq}\rho_{rs} - \frac{1}{2}\rho_{rq}\rho_{ps}\right) \tag{46}$$

is obtained from the 2-RDM matrix element given as the expectation value

$$\Gamma^{pr}_{qs} = \left\langle \Psi^0 \middle| e^{S^\dagger} e^{-T}(E_{pq}E_{rs} - \delta_{rq}E_{ps})e^{T} e^{-S^\dagger} \middle| \Psi^0 \right\rangle \tag{47}$$

The antisymmetrized product of 1-RDMs on the rhs of Eq. 46 cancels out the disconnected terms in Γ^{pr}_{qs}. After insertion of Eqs. 43-45 into Eq. 46 and software-assisted evaluation, the permutationally unique elements of $\Lambda^{\text{CCSD[2]}}$ are as follows:

$$(\Lambda^{\text{CCD[2]}})^{ab}_{ij} = (\Lambda^{\text{CCSD[2]}})^{ab}_{ij} = 4T^{ab}_{ij} - 2T^{ab}_{ji} \tag{48}$$

$$(\Lambda^{\text{CCD[2]}})^{aj}_{ib} = (\Lambda^{\text{CCSD[2]}})^{aj}_{ib} = \sum_{ck}^{\text{orb}} \left(8T^{ac}_{ik}T^{cb}_{kj} - 4T^{ac}_{ik}T^{cb}_{jk} - 4T^{ac}_{ki}T^{cb}_{kj} + 2T^{ac}_{ki}T^{cb}_{jk}\right) \tag{49}$$

$$(\Lambda^{\text{CCD[2]}})^{ik}_{jl} = (\Lambda^{\text{CCSD[2]}})^{ik}_{jl} = \sum_{ab}^{\text{orb}} \left(4T^{ab}_{ik}T^{ba}_{lj} - 2T^{ab}_{ik}T^{ba}_{jl}\right) \tag{50}$$

$$(\Lambda^{\text{CCD[2]}})^{ia}_{jb} = (\Lambda^{\text{CCSD[2]}})^{ia}_{jb} = \sum_{ck}^{\text{orb}} \left(-4T^{ac}_{jk}T^{cb}_{ki} - 4T^{ac}_{kj}T^{cb}_{ik} + 2T^{ac}_{jk}T^{cb}_{ik} + 2T^{ac}_{kj}T^{cb}_{ki}\right) \tag{51}$$

$$(\Lambda^{\text{CCD[2]}})^{ac}_{bd} = (\Lambda^{\text{CCSD[2]}})^{ac}_{bd} = \sum_{ij}^{\text{orb}} \left(4T^{ac}_{ij}T^{db}_{ji} - 2T^{ac}_{ij}T^{db}_{ij}\right) \tag{52}$$

$$(\Lambda^{\text{CCSD[2]}})^{ab}_{ic} = \sum_{j}^{\text{orb}} \left(4T^{ab}_{ij}T_{cj} - 2T^{ab}_{ji}T_{cj}\right) \tag{53}$$

$$(\Lambda^{\text{CCSD[2]}})^{aj}_{ik} = \sum_{b}^{\text{orb}} \left(-4T^{ab}_{ik}T_{bj} + 2T^{ab}_{ki}T_{bj}\right) \tag{54}$$

The remaining contributions to Λ can be obtained from the permutation relations

$$\Lambda^{pr}_{qs} = \Lambda^{rp}_{sq} \tag{55}$$

$$\Lambda^{pr}_{qs} = \Lambda^{qs}_{pr} \tag{56}$$

The matrix elements in Eqs. 48-54 and Eqs. 43-45 satisfy the trace condition of the cumulant[S24,S25] up to the second order of MBPT

$$\sum_{r}^{\text{orb}}(\Lambda^{(m)})_{qr}^{pr} = \frac{1}{2}\sum_{n=0}^{m}\left(\rho^{(\text{m-n})}\rho^{(\text{n})}\right)_{pq} - \rho_{pq}^{(\text{m})} \quad \text{for } m = 0, 1, 2 \tag{57}$$

where the upper index in parentheses (m) denotes mth-order contribution in the MBPT expansion. $\Lambda^{\text{CCSD[2]}}$ agrees with the exact cumulant through the second order of MBPT if evaluated with sufficiently accurate amplitudes. However, in the RPA approach, T_2 is only accurate through the first order, which limits the accuracy of the linear contribution to $\Lambda^{\text{CCD[2]}}$. The leading-order role of T_1 in $\rho^{\text{CCSD[2]}}$ explains our interpretation of $E_{\text{c}}^{\text{1RDM}}$ as the beyond-RPA term accounting for single excitations.

4.3 RPA auxiliary amplitudes

The RPA formulas for 1-RDM and the cumulant require the infinite-order ring approximation of S, which can be obtained by solving Eq. 39 with only ring contributions retained on the rhs. Let us restrict S to double excitations, i.e., $S = S_2$. Figure S1 shows the direct-ring diagrams[S15] contributing to the S_2 amplitude equation. The corresponding matrix equation

$$S_2 = T_2 - 4(T_2)^3 + 4S_2(T_2)^2 + 4(T_2)^2 S_2 - 16(T_2)^2 S_2 (T_2)^2 \tag{58}$$

has the closed-form solution

$$S_2 = T_2/(1 - 4(T_2)^2) \tag{59}$$

Eq. 59 is the infinite-order ring (RPA) approximation of S.

4.4 RPA+X approximation of the coupled-cluster doubles 1-RDM

We will derive the infinite-order ring approximation (RPA) of 1-RDM, corrected by the quadratic-doubles exchange terms from Eqs. 44 and 45. Separating the ring contributions

Table S2: Direct-ring (RPA) coupled-cluster doubles contributions to the correlated part of 1-RDM. The operators T_2 and S_2 are represented by the solid and curly lines, respectively.

direct-ring contribution	closed-shell expression
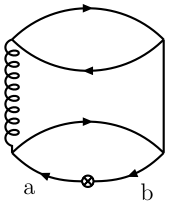	$\delta\rho_{ab}^{\mathrm{RPA}} = 4\sum_i^{\mathrm{orb}}(S_2T_2)_{ii}^{ab}$
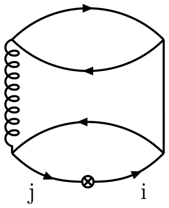	$\delta\rho_{ij}^{\mathrm{RPA}} = -4\sum_a^{\mathrm{orb}}(T_2S_2)_{ij}^{aa}$

on the rhs of Eq. 42 and inserting the ring approximation of S_2 yields the 1-RDM at the RPA level. The diagrams contributing to $\delta\rho^{\mathrm{RPA}}$ and the corresponding algebraic formulas are listed in Table S2. The *ov* and *vo* parts of $\delta\rho^{\mathrm{RPA}}$ are zero because of the lack of single excitations. In the lowest order, the auxiliary amplitudes can be approximated as $S_2 \approx T_2$. Thus, $\delta\rho^{\mathrm{RPA}}$ recovers the direct, doubles-only part $\rho^{\mathrm{CCSD[2]}}$. The 1-RDM analogue of the second-order exchange correlation-energy correction can be formed by adding the doubles exchange part of $\delta\rho^{[2]}$, Eqs. 44 and 45, to the *oo* and *vv* blocks of $\delta\rho^{\mathrm{RPA}}$. This defines the RPA+X approximation of the correlated part of 1-RDM:

$$\delta\rho_{ij}^{\mathrm{RPA+X}} = \sum_{abk}^{\mathrm{orb}} \left(-4S_{ik}^{ab}T_{kj}^{ba} + 2T_{ik}^{ab}T_{jk}^{ba}\right) \tag{60}$$

$$\delta\rho_{ab}^{\mathrm{RPA+X}} = \sum_{cij}^{\mathrm{orb}} \left(4T_{ij}^{ac}S_{ji}^{cb} - 2T_{ij}^{ac}T_{ij}^{cb}\right) \tag{61}$$

The RPA+X approximation is not useful per se because of the lack of the leading singles contribution but will be essential for the derivation of the beyond-RPA corrections to the coupled-cluster expectation value of the full hamiltonian in Section 5.

4.5 RPA cumulant matrix

The infinite-order ring approximation of the cumulant, Λ^{RPA}, is the sum of all connected ring terms contributing to Γ^{pr}_{qs} on the rhs of Eq. 47. The individual ring diagrams and the corresponding algebraic expressions are listed in Table S3. As Λ^{RPA} always appears in contractions with the Coulomb matrix, it is useful to define an auxiliary matrix which gathers all contributions of Λ^{RPA} multiplying a permutationally-unique Coulomb integral $(ai|bj)$:

$$(\overline{\Lambda^{\mathrm{RPA}}})^{ab}_{ij} = (\Lambda^{\mathrm{RPA}})^{ab}_{ij} + (\Lambda^{\mathrm{RPA}})^{ib}_{aj} + (\Lambda^{\mathrm{RPA}})^{aj}_{ib} + (\Lambda^{\mathrm{RPA}})^{ij}_{ab} = 2(\Lambda^{\mathrm{RPA}})^{ab}_{ij} + 2(\Lambda^{\mathrm{RPA}})^{aj}_{ib} \tag{62}$$

Substituting the algebraic expressions from Table S3 results in

$$\overline{\Lambda^{\mathrm{RPA}}} = 8T_2/(1-2T_2) \tag{63}$$

which, after substitution of T_2 from Eq. 1, assumes a particularly simple form

$$\overline{\Lambda^{\mathrm{RPA}}} = 4\mathcal{I} = -\frac{1}{\pi}\int_0^\infty \mathrm{d}u \left(\chi^{\mathrm{RPA}}(u) - \chi(u)\right) \tag{64}$$

5 Beyond-RPA terms in the expectation value of H

Consider the coupled-cluster doubles (CCD) correlation energy defined as the expectation value of the full normal-ordered hamiltonian

$$E_{\mathrm{c}}^{\mathrm{CCD}} = \frac{\left\langle e^{T_2}\Psi^0\right| H \left|e^{T_2}\Psi^0\right\rangle}{\langle e^{T_2}\Psi^0|e^{T_2}\Psi^0\rangle} - \left\langle \Psi^0\right| H \left|\Psi^0\right\rangle = \frac{\left\langle e^{T_2}\Psi^0\right| H_N \left|e^{T_2}\Psi^0\right\rangle}{\langle e^{T_2}\Psi^0|e^{T_2}\Psi^0\rangle} \tag{65}$$

with the cluster operator T_2 being the solution of the RPA doubles equation. Wigner's $2n+1$ rule[S19,S26] applies to Eq. 65, which means that using $\overline{\Lambda^{\mathrm{RPA}}}$, $\rho^{\mathrm{RPA+X}}$, and the non-ring terms

Table S3: Contributions to the RPA cumulant. The operators T_2 and S_2 are represented by the solid and curly lines, respectively. The dotted line denotes a two-electron operator X.

direct-ring contribution	closed-shell expression
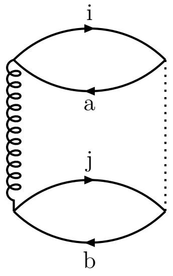	$(\Lambda^{\mathrm{RPA}})^{ab}_{ij} = 4S^{ab}_{ij} = (4T_2/(1-4(T_2)^2))^{ab}_{ij}$
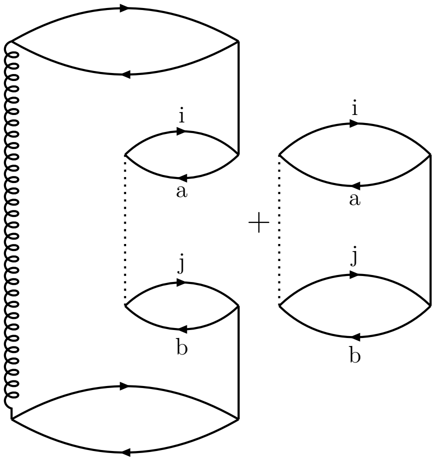	$(\Lambda^{\mathrm{RPA}})^{ij}_{ab} = (16T_2S_2T_2 + 4T_2)^{ab}_{ij} = (\Lambda^{\mathrm{RPA}})^{ab}_{ij}$
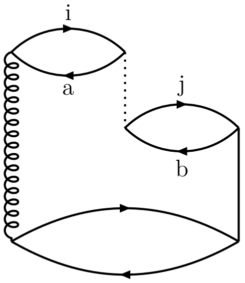	$(\Lambda^{\mathrm{RPA}})^{aj}_{ib} = (8S_2T_2)^{ab}_{ij} = (8(T_2)^2/(1-4(T_2)^2))^{ab}_{ij}$
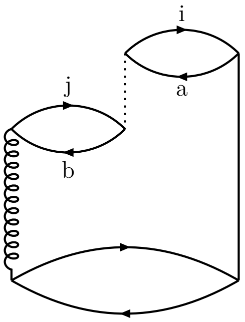	$(\Lambda^{\mathrm{RPA}})^{ib}_{aj} = (8S_2T_2)^{ab}_{ij} = (\Lambda^{\mathrm{RPA}})^{aj}_{ib}$

from $\Lambda^{\text{CCD[2]}}$ is sufficient to recover the CCD correlation energy through third order

$$E_{\text{c}}^{\text{CCD}} = \text{Tr}\left(h\delta\rho^{\text{RPA+X}}\right) + \frac{1}{2}\,\text{Tr}\left(\overline{\Lambda^{\text{RPA}}}V\right) + \frac{1}{2}\sum_{pqrs}^{\text{orb}}(\Delta\Lambda^{\text{CCD[2]}})^{pr}_{qs}(pq|rs) + \text{non-ring }\mathcal{O}(\lambda^4) \quad (66)$$

where $\Delta\Lambda^{\text{CCD[2]}}$ denotes the quadratic CCD cumulant matrix elements from Eqs. 48-52 with all direct-ring terms subtracted

$$(\Delta\Lambda^{\text{CCD[2]}})^{ab}_{ij} = -2(T)^{ab}_{ji} \quad (67)$$

$$(\Delta\Lambda^{\text{CCD[2]}})^{aj}_{ib} = \sum_{ck}^{\text{orb}}\left(-4(T)^{ac}_{ik}(T)^{cb}_{jk} - 4(T)^{ac}_{ki}(T)^{cb}_{kj} + 2(T)^{ac}_{ki}(T)^{cb}_{jk}\right) \quad (68)$$

$$(\Delta\Lambda^{\text{CCD[2]}})^{ik}_{jl} = (\Lambda^{\text{CCD[2]}})^{ik}_{jl} \qquad (\Delta\Lambda^{\text{CCD[2]}})^{ia}_{jb} = (\Lambda^{\text{CCD[2]}})^{ia}_{jb} \qquad (\Delta\Lambda^{\text{CCD[2]}})^{ac}_{bd} = (\Lambda^{\text{CCD[2]}})^{ac}_{bd} \quad (69)$$

The antisymmetrized 1-RDM term, $E_{\text{c}}^{\text{1RDM,quad}}$, does not appear in Eq. 66 because it starts contributing in the fifth order.

The one-electron part of $E_{\text{c}}^{\text{CCD}}$ depends only on the zeroth-order part of the hamiltonian, h^0, because the one-electron perturbation in the GMBPT hamiltonian has zero oo and vv contributions. Hence, the one-electron part of Eq. 66 can be rewritten as

$$\begin{aligned}\text{Tr}\left(h\rho^{\text{RPA+X}}\right) &= \text{Tr}\left(h^0\rho^{\text{RPA+X}}\right)\\ &= \sum_{ab}^{\text{orb}}\delta\rho^{\text{RPA+X}}_{ab}h^0_{ab} + \sum_{ij}^{\text{orb}}\delta\rho^{\text{RPA+X}}_{ij}h^0_{ij}\\ &= 4\sum_{aibj}^{\text{orb}}S^{ab}_{ij}T^{ab}_{ij}(\epsilon_a - \epsilon_i) - 2\sum_{aibj}^{\text{orb}}T^{ab}_{ji}T^{ab}_{ij}(\epsilon_a - \epsilon_i)\end{aligned} \quad (70)$$

We will eliminate the orbital energy differences from Eq. 70 in order to obtain the RPA correlation energy and beyond-RPA corrections as explicit functions of T_2 and V. Multiplying the rhs of the RPA doubles equation, Eq. 3, by S^{ab}_{ij} and T^{ab}_{ji} and summing over all free indices

results in

$$\sum_{aibj}^{\text{orb}} S_{ij}^{ab} T_{ij}^{ab} (\epsilon_a - \epsilon_i) = -\frac{1}{2} \operatorname{Tr}\left((S_2 + 4S_2T_2 + 4T_2S_2T_2)V \right) \\ = -\frac{1}{2} \left(\frac{T_2 + 4T_2^2 + 4T_2^3}{1 - 4T_2^2} V \right) \\ = -\frac{1}{8} \operatorname{Tr}\left(\overline{\Lambda^{\text{RPA}}} V \right) + \frac{1}{2} \operatorname{Tr}(T_2 V) \tag{71}$$

and

$$\sum_{aibj}^{\text{orb}} T_{ji}^{ab} T_{ij}^{ab} (\epsilon_a - \epsilon_i) = -\frac{1}{2} \sum_{aibj}^{\text{orb}} T_{ji}^{ab} (ai|bj) - 2 \sum_{aibjck}^{\text{orb}} T_{ki}^{ac} T_{kj}^{cb} (ai|bj) \\ - 2 \sum_{aibjckdl}^{\text{orb}} T_{ik}^{ac} T_{lk}^{cd} T_{lj}^{db} (ai|bj) \\ = \frac{1}{4} E_{\text{c}}^{1b} + \frac{1}{4} (E_{\text{c}}^{2b} + E_{\text{c}}^{2c}) \\ - 2 \sum_{aibjckdl}^{\text{orb}} T_{ik}^{ac} T_{lk}^{cd} T_{lj}^{db} (ai|bj) \tag{72}$$

where we have identified the RPA cumulant $\overline{\Lambda^{\text{RPA}}}$ and the non-ring cumulant energy contributions E_{c}^{1b}, E_{c}^{2b}, and E_{c}^{2c} listed in Table S4. Substitution of Eqs. 71 and 72 back into Eq. 66 and using the cumulant energy terms specified in Table S4 yields the expansion

$$E_{\text{c}}^{\text{CCD}} = 2 \operatorname{Tr}(T_2 V) + \frac{1}{2} E_{\text{c}}^{1b} + \frac{1}{2} (E_{\text{c}}^{2b} + E_{\text{c}}^{2c}) + E_{\text{c}}^{2d} + \ldots + E_{\text{c}}^{2l} + \text{non-ring } \mathcal{O}(\lambda^4) \tag{73}$$

The above equation is the central result of this work from which we derive the third-order beyond-RPA corrections in terms of T_2. The first term on the rhs of Eq. 73 is equal to the direct-ring RPA correlation energy[S1]

$$E_{\text{c}}^{\text{RPA}} = 2 \operatorname{Tr}(T_2 V) \tag{74}$$

and the second term is the SOSEX correction[S27]

$$E_{\mathrm{c}}^{\mathrm{SOSEX}} = \frac{1}{2} E_{\mathrm{c}}^{1b} \tag{75}$$

Therefore, $E_{\mathrm{c}}^{\mathrm{SOSEX}}$ and the remaining non-ring terms of Eq. 73 define the beyond-RPA correlation energy correction at the quadratic CCD level

$$\Delta E_{\mathrm{c}}^{\mathrm{CCD[2]}} = E_{\mathrm{c}}^{\mathrm{SOSEX}} + \frac{1}{2}(E_{\mathrm{c}}^{2b} + E_{\mathrm{c}}^{2c}) + E_{\mathrm{c}}^{2d} + \ldots + E_{\mathrm{c}}^{2l} \tag{76}$$

where all pure doubles MBPT contributions through third order are included exactly.

Taking into account the complete set of terms from $\Delta E_{\mathrm{c}}^{\mathrm{CCD[2]}}$ requires CPU and memory resources significantly above the usual cost of RPA computations. For this reason, only two least expensive terms from Eq. 76, namely, $E_{\mathrm{c}}^{\mathrm{SOSEX}}$ and E_{c}^{2g}, are retained in the practical variant. Those beyond-RPA doubles contributions are supplemented by the mean-field model of $E_{\mathrm{c}}^{\mathrm{1RDM,lin}}$ and $E_{\mathrm{c}}^{\mathrm{1RDM,quad}}$ to account for the leading singles contribution to the expectation value of H. The resulting total correlation energy

$$E_{\mathrm{c}} = E_{\mathrm{c}}^{\mathrm{RPA}} + E_{\mathrm{c}}^{\mathrm{1RDM,lin}} + E_{\mathrm{c}}^{\mathrm{1RDM,quad}} + E_{\mathrm{c}}^{\mathrm{SOSEX}} + E_{\mathrm{c}}^{2g} \tag{77}$$

is the final, quartic-scaling approximation applied in all numerical tests presented in this work.

6 Adiabatic connection

In Section 5 we have considered only the fully interacting system ($\lambda = 1$). It might be tempting to approach the same derivation using the adiabatic connection (AC) technique,[S18,S28] which avoids the need to compute the expectation value of the zeroth order hamiltonian. However, we will show that the AC technique does not recover the exact third-order non-ring

Table S4: Linear and quadratic contributions to the cumulant part of the coupled-cluster doubles expectation value of the hamiltonian. Numerical prefactors related to the permutational symmetry of Λ^{pr}_{qs} are indicated in the first column in front of each summation symbol. The quadratic CCD approximation of the cumulant matrix is denoted as $\Lambda^{\mathrm{CCD[2]}}$.

trace contribution	intermediates
$2 \times \frac{1}{2} \sum_{aibj}^{\mathrm{orb}} (ai\|bj)(\Lambda^{\mathrm{CCD[2]}})^{ab}_{ij} = E_{\mathrm{c}}^{1a} + E_{\mathrm{c}}^{1b}$	$E_{\mathrm{c}}^{1a} = 4 \sum_{aibj}^{\mathrm{orb}} (ai\|bj) T^{ab}_{ij}$ $E_{\mathrm{c}}^{1b} = -2 \sum_{aibj}^{\mathrm{orb}} (ai\|bj) T^{ab}_{ji}$
$2 \times \frac{1}{2} \sum_{aibj}^{\mathrm{orb}} (ai\|jb)(\Lambda^{\mathrm{CCD[2]}})^{aj}_{ib} = E_{\mathrm{c}}^{2a} + E_{\mathrm{c}}^{2b} + E_{\mathrm{c}}^{2c} + E_{\mathrm{c}}^{2d}$	$E_{\mathrm{c}}^{2a} = 8 \sum_{aibjck}^{\mathrm{orb}} (ai\|jb) T^{ac}_{ik} T^{cb}_{kj}$ $E_{\mathrm{c}}^{2b} = -4 \sum_{aibjck}^{\mathrm{orb}} (ai\|jb) T^{ac}_{ik} T^{cb}_{jk}$ $E_{\mathrm{c}}^{2c} = -4 \sum_{aibjck}^{\mathrm{orb}} (ai\|jb) T^{ac}_{ki} T^{cb}_{kj}$ $E_{\mathrm{c}}^{2d} = 2 \sum_{aibjck}^{\mathrm{orb}} (ai\|jb) T^{ac}_{ki} T^{cb}_{jk}$
$\frac{1}{2} \sum_{ijkl}^{\mathrm{orb}} (ij\|kl)(\Lambda^{\mathrm{CCD[2]}})^{ik}_{jl} = E_{\mathrm{c}}^{2e} + E_{\mathrm{c}}^{2f}$	$E_{\mathrm{c}}^{2e} = 2 \sum_{aibjkl}^{\mathrm{orb}} (ij\|kl) T^{ab}_{ik} T^{ba}_{lj}$ $E_{\mathrm{c}}^{2f} = - \sum_{aibjkl}^{\mathrm{orb}} (ij\|kl) T^{ab}_{ik} T^{ba}_{jl}$
$2 \times \frac{1}{2} \sum_{aibj}^{\mathrm{orb}} (ij\|ab)(\Lambda^{\mathrm{CCD[2]}})^{ia}_{jb} = E_{\mathrm{c}}^{2g} + E_{\mathrm{c}}^{2h} + E_{\mathrm{c}}^{2i} + E_{\mathrm{c}}^{2j}$	$E_{\mathrm{c}}^{2g} = -4 \sum_{aibjck}^{\mathrm{orb}} (ij\|ab) T^{ac}_{jk} T^{cb}_{ki}$ $E_{\mathrm{c}}^{2h} = -4 \sum_{aibjck}^{\mathrm{orb}} (ij\|ab) T^{ac}_{kj} T^{cb}_{ik}$ $E_{\mathrm{c}}^{2i} = 2 \sum_{aibjck}^{\mathrm{orb}} (ij\|ab) T^{ac}_{jk} T^{cb}_{ik}$ $E_{\mathrm{c}}^{2j} = 2 \sum_{aibjck}^{\mathrm{orb}} (ij\|ab) T^{ac}_{kj} T^{cb}_{ki}$
$\frac{1}{2} \sum_{abcd}^{\mathrm{orb}} (ab\|cd)(\Lambda^{\mathrm{CCD[2]}})^{ac}_{bd} = E_{\mathrm{c}}^{2k} + E_{\mathrm{c}}^{2l}$	$E_{\mathrm{c}}^{2k} = 2 \sum_{aibjcd}^{\mathrm{orb}} (ab\|cd) T^{ac}_{ij} T^{db}_{ji}$ $E_{\mathrm{c}}^{2l} = - \sum_{aibjcd}^{\mathrm{orb}} (ab\|cd) T^{ac}_{ij} T^{db}_{ij}$

terms if applied with the RPA doubles, as opposed to the $\lambda = 1$ approach.

In the AC approach, we express the correlation energy as the coupling-constant integral of the expectation value of the perturbation $\delta H_N = H_N - H_N^0$

$$E_{\rm c} = \int_0^1 \, {\rm d}\lambda \frac{\left\langle \Psi^\lambda \middle| \, \delta H_N \, \middle| \Psi^\lambda \right\rangle}{\left\langle \Psi^\lambda \middle| \Psi^\lambda \right\rangle} \tag{78}$$

where $\left|\Psi^\lambda\right\rangle$ is the ground-state eigenvector of the linear-switching AC hamiltonian

$$H^\lambda \left|\Psi^\lambda\right\rangle = E_{\rm tot}^\lambda \left|\Psi^\lambda\right\rangle \tag{79}$$

$$H^\lambda = (1-\lambda)H^0 + \lambda H \tag{80}$$

All previously derived expressions in the expectation-value coupled-cluster formalism can be re-used for the integrand in Eq. 78, except now T_2^λ originates from the λ-dependent amplitude equation

$$\begin{aligned} 0 = {} & \lambda(ai|bj) + (T^\lambda)_{ij}^{ab}(\epsilon_a + \epsilon_b - \epsilon_i - \epsilon_j) + 2\lambda \sum_{ck}^{\rm orb} (T^\lambda)_{ik}^{ac}(ck|bj) \\ & + 2\lambda \sum_{ck}^{\rm orb} (ai|ck)(T^\lambda)_{kj}^{cb} + 4\lambda \sum_{ckdl}^{\rm orb} (T^\lambda)_{ik}^{ac}(ck|dl)(T^\lambda)_{lj}^{db} \end{aligned} \tag{81}$$

Analogously to Eq. 64, the RPA cumulant corresponding to $\left|\Psi^\lambda\right\rangle$ is given by

$$\overline{\Lambda^{\rm RPA}}(\lambda) = 8T_2^\lambda/(1-2T_2^\lambda) \tag{82}$$

which can be rewritten as

$$\overline{\Lambda^{\rm RPA}}(\lambda) = -\frac{1}{\pi}\int_0^\infty \, {\rm d}u \left(\chi^{\rm RPA}(u;\lambda) - \chi(u)\right) \tag{83}$$

using the RPA response function, $\chi^{\mathrm{RPA}}(u;\lambda)$, from the λ-dependent screening equation

$$\chi^{\mathrm{RPA}} = \chi + \chi\lambda v\chi^{\mathrm{RPA}} \tag{84}$$

Now let us consider only the ring part of Eq. 78. After substitution of Eq. 83, the AC integral simplifies to

$$\begin{aligned} E_{\mathrm{c}}^{\mathrm{RPA}} &= \int_0^1 \mathrm{d}\lambda\ \mathrm{Tr}\left(\overline{\Lambda^{\mathrm{RPA}}}(\lambda)V\right) \\ &= -\frac{1}{2\pi}\int_0^1 \mathrm{d}\lambda \int_0^\infty \mathrm{d}u\,\mathrm{Tr}\left((\chi^{\mathrm{RPA}}(u;\lambda)-\chi(u))V\right) \end{aligned} \tag{85}$$

which is exactly equivalent to the the RPA correlation energy in the coupled-cluster formulation[S1] as given by Eq. 74. However, the beyond-RPA corrections are different from those derived in the $\lambda = 1$ approach. Assuming the CCD parametrization of Eq. 78 with RPA doubles, the AC integral expands into

$$\begin{aligned} \int_0^1 \mathrm{d}\lambda \frac{\left\langle e^{T_2^\lambda}\Psi^0 \middle| \delta H_N \middle| e^{T_2^\lambda}\Psi^0 \right\rangle}{\left\langle e^{T_2^\lambda}\Psi^0 \middle| e^{T_2^\lambda}\Psi^0 \right\rangle} &= E_{\mathrm{c}}^{\mathrm{RPA}} + \int_0^1 \mathrm{d}\lambda \Big(E_{\mathrm{c}}^{1b}\left[T_2^\lambda\right] + E_{\mathrm{c}}^{2b}\left[T_2^\lambda\right] + E_{\mathrm{c}}^{2c}\left[T_2^\lambda\right] + \ldots \\ &\ldots + E_{\mathrm{c}}^{2l}\left[T_2^\lambda\right] \Big) + \text{non-ring 4th- and higher-order terms} \end{aligned} \tag{86}$$

In the sum of terms enclosed by parentheses, the second-order contribution to $E_{\mathrm{c}}^{\mathrm{SOSEX}}$ is recovered, but none of the third-order contributions has the exact MBPT coefficient, e.g., the 2g contribution acquires an incorrect factor of 1/3 due to the λ integration. This is an example of a general rule: an nth order wave function is sufficiently accurate to obtain the expectation value of the total hamiltonian through order $2n+1$,[S26] as in Eq. 65, but the expectation value of the perturbation, as in Eq. 78, can only be accurate through order $n+1$.

References

(S1) Scuseria, G. E.; Henderson, T. M.; Sorensen, D. C. The ground state correlation energy of the random phase approximation from a ring coupled cluster doubles approach. *J. Chem. Phys.* **2008**, *129*, 231101.

(S2) Eshuis, H.; Yarkony, J.; Furche, F. Fast computation of molecular random phase approximation correlation energies using resolution of the identity and imaginary frequency integration. *J. Chem. Phys.* **2010**, *132*, 234114.

(S3) Hager, W. W. Updating the inverse of a matrix. *SIAM Rev.* **1989**, *31*, 221–239.

(S4) Higham, N. J. *Functions of Matrices*; Society for Industrial and Applied Mathematics, 2008.

(S5) Parrish, R. M.; Zhao, Y.; Hohenstein, E. G.; Martínez, T. J. Rank reduced coupled cluster theory. I. Ground state energies and wavefunctions. *J. Chem. Phys.* **2019**, *150*, 164118.

(S6) Parrish, R. M.; Hohenstein, E. G.; Sherrill, C. D. Tractability gains in symmetry-adapted perturbation theory including coupled double excitations: CCD+ST(CCD) dispersion with natural orbital truncations. *J. Chem. Phys.* **2013**, *139*, 174102.

(S7) Lesiuk, M. Quintic-scaling rank-reduced coupled cluster theory with single and double excitations. *J. Chem. Phys.* **2022**, *156*, 064103.

(S8) Hoja, J.; Ko, H.-Y.; Neumann, M. A.; Car, R.; DiStasio, R. A.; Tkatchenko, A. Reliable and practical computational description of molecular crystal polymorphs. *Sci. Adv.* **2019**, *5*, eaau3338.

(S9) Halko, N.; Martinsson, P.-G.; Tropp, J. A. Finding structure with randomness: Probabilistic algorithms for constructing approximate matrix decompositions. *SIAM Rev.* **2011**, *53*, 217–288.

(S10) Modrzejewski, M.; Yourdkhani, S.; Klimes, J. Random Phase Approximation Applied to Many-Body Noncovalent Systems. *J. Chem. Theory Comput.* **2020**, *16*, 427–442.

(S11) Modrzejewski, M.; Yourdkhani, S.; Śmiga, S.; Klimes, J. Random-phase approximation in many-body noncovalent systems: Methane in a dodecahedral water cage. *J. Chem. Theory Comput.* **2021**, *17*, 804–817.

(S12) Ren, X.; Rinke, P.; Scuseria, G. E.; Scheffler, M. Renormalized second-order perturbation theory for the electron correlation energy: Concept, implementation, and benchmarks. *Phys. Rev. B* **2013**, *88*, 035120.

(S13) Bartlett, R. J. Ab initio DFT and its role in electronic structure theory. *Mol. Phys.* **2010**, *108*, 3299–3311.

(S14) Paldus, J.; Čížek, J. In *Time-Independent Diagrammatic Approach to Perturbation Theory of Fermion Systems*; Löwdin, P.-O., Ed.; Adv. Quantum Chem.; Academic Press, 1975; Vol. 9; pp 105–197.

(S15) Paldus, J. In *Springer Handbook of Atomic, Molecular, and Optical Physics*; Drake, G. W. F., Ed.; Springer International Publishing: Cham, 2023; pp 95–109.

(S16) Verma, P.; Bartlett, R. J. Increasing the applicability of density functional theory. II. Correlation potentials from the random phase approximation and beyond. *J. Chem. Phys.* **2012**, *136*, 044105.

(S17) Bartlett, R. J.; Grabowski, I.; Hirata, S.; Ivanov, S. The exchange-correlation potential in ab initio density functional theory. *J. Chem. Phys.* **2005**, *122*, 034104.

(S18) Klimes, J.; Kaltak, M.; Maggio, E.; Kresse, G. Singles correlation energy contributions in solids. *J. Chem. Phys.* **2015**, *143*, 102816.

(S19) Jeziorski, B.; Moszyński, R. Explicitly connected expansion for the average value of an observable in the coupled-cluster theory. *Int. J. Quant. Chem.* **1993**, *48*, 161–183.

(S20) Arponen, J. Variational principles and linked-cluster exp S expansions for static and dynamic many-body problems. *Ann. Phys.* **1983**, *151*, 311–382.

(S21) Trucks, G. W.; Salter, E.; Sosa, C.; Bartlett, R. J. Theory and implementation of the MBPT density matrix. An application to one-electron properties. *Chem. Phys. Lett.* **1988**, *147*, 359–366.

(S22) Korona, T. Two-particle density matrix cumulant of coupled cluster theory. *Phys. Chem. Chem. Phys.* **2008**, *10*, 5698–5705.

(S23) Korona, T. First-order exchange energy of intermolecular interactions from coupled cluster density matrices and their cumulants. *J. Chem. Phys.* **2008**, *128*, 224104.

(S24) Kutzelnigg, W.; Mukherjee, D. Cumulant expansion of the reduced density matrices. *J. Chem. Phys.* **1999**, *110*, 2800–2809.

(S25) Mukherjee, D.; Kutzelnigg, W. Irreducible Brillouin conditions and contracted Schrödinger equations for n-electron systems. I. The equations satisfied by the density cumulants. *J. Chem. Phys.* **2001**, *114*, 2047–2061.

(S26) Mayer, I. *Simple theorems, proofs, and derivations in quantum chemistry*; Springer New York, NY, 2003.

(S27) Grüneis, A.; Marsman, M.; Harl, J.; Schimka, L.; Kresse, G. Making the random phase approximation to electronic correlation accurate. *J. Chem. Phys.* **2009**, *131*, 154115.

(S28) Pernal, K. Exact and approximate adiabatic connection formulae for the correlation energy in multireference ground and excited states. *J. Chem. Phys.* **2018**, *149*, 204101.
